# Fluctuation Relations to Calculate Protein Redox Potentials from Molecular Dynamics Simulations

**DOI:** 10.1021/acs.jctc.3c00785

**Published:** 2023-12-27

**Authors:** A. S. F. Oliveira, J. Rubio, C. E. M. Noble, J. L. R. Anderson, J. Anders, A. J. Mulholland

**Affiliations:** † Centre for Computational Chemistry, School of Chemistry, University of Bristol, Bristol BS8 1TS, U.K.; ‡ School of Biochemistry, University of Bristol, Bristol BS8 1DT, U.K.; § BrisSynBio Synthetic Biology Research Centre, University of Bristol, Bristol BS8 1TQ, U.K.; ∥ School of Mathematics and Physics, University of Surrey, Guildford GU2 7XH, U.K.; ⊥ Department of Physics and Astronomy, University of Exeter, Stocker Road, Exeter EX4 4QL, U.K.; # Institute of Physics and Astronomy, University of Potsdam, Potsdam 14476, Germany

## Abstract

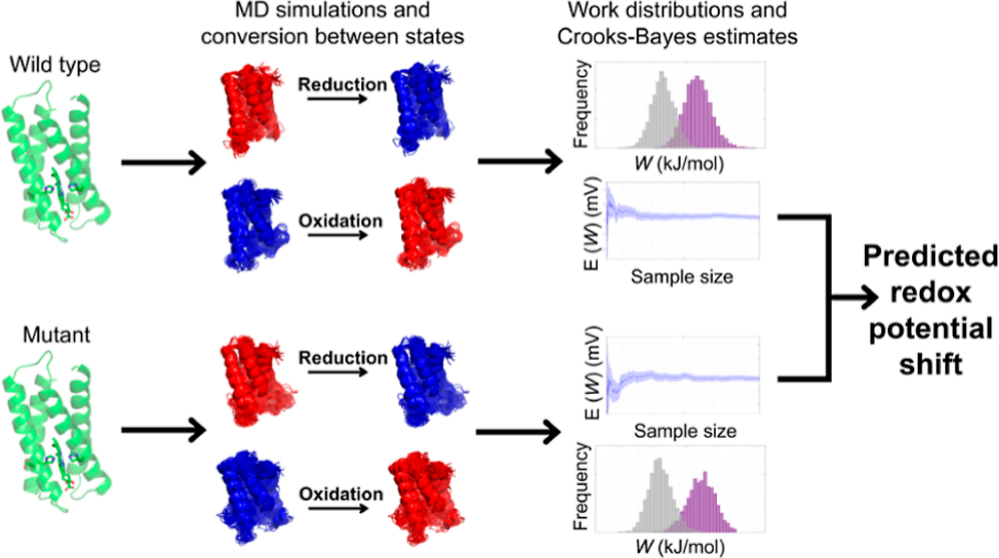

The tunable design of protein redox potentials promises to open a range of applications in biotechnology and catalysis. Here, we introduce a method to calculate redox potential changes by combining fluctuation relations with molecular dynamics simulations. It involves the simulation of reduced and oxidized states, followed by the instantaneous conversion between them. Energy differences introduced by the perturbations are obtained using the Kubo-Onsager approach. Using a detailed fluctuation relation coupled with Bayesian inference, these are postprocessed into estimates for the redox potentials in an efficient manner. This new method, denoted MD + CB, is tested on a *de novo* four-helix bundle heme protein (the m4D2 “maquette”) and five designed mutants, including some mutants characterized experimentally in this work. The MD + CB approach is found to perform reliably, giving redox potential shifts with reasonably good correlation (0.85) to the experimental values for the mutants. The MD + CB approach also compares well with redox potential shift predictions using a continuum electrostatic method. The estimation method employed within the MD + CB approach is straightforwardly transferable to standard equilibrium MD simulations and holds promise for redox protein engineering and design applications.

## 1 Introduction

Electron transfer is fundamental in biological processes such as photosynthesis and respiration. Evolution has modulated the redox properties of proteins involved in redox processes to make electron transfer rates sufficient to sustain such processes.^1−4^ Redox-active metallocofactors, such as heme, enable many natural oxidoreductases to catalyze a wide range of reactions, including hydroxylation and oxygenation.^1,5^ Heme-containing proteins are ubiquitously found in nature and are involved in many biological electron transfer processes.^1,6^ Their redox potentials play a role in determining their activities, such as oxygen binding, electron transfer, and catalysis.^1,6^

The redox potential *E* of a heme center can be described as its tendency to acquire electrons and, thus, to become reduced. Thus, the higher the value of the redox potential, the more favorable the reduction of the group. The intrinsic properties of the heme macrocycle are critically modulated by the protein environment.^1,6^ Many properties are important for this, including the axial residues directly coordinating the iron, the second coordination sphere interactions, such as hydrogen bonds, and the electrostatic environment surrounding the center.^1,6^ Given the multitude of factors involved in this “tuning”, the accurate prediction of the redox properties of a heme-containing protein remains a challenge. There is a need for computational methods capable of predicting redox potentials for natural proteins (*e.g.*, for analyzing the effects of mutations) and potentially in the engineering of proteins, both natural and *de novo*, with altered redox properties.

Engineering existing redox proteins and the construction of novel designs offer possibilities such as tuning enzymes toward alternative substrates and creating novel electron transfer systems^3,7^ for applications in biocatalysis, biosensing, biofuel generation, and bioelectronics.^6,8^ Reliable prediction methods will assist functional protein design and complement directed evolution by identifying target sites for mutation. Calculations of the redox properties of mutant proteins could usefully be incorporated into design protocols, for example, to identify promising locations for mutations for synthesis and narrow the experimental search spaces for desired properties.

Despite difficulties, examples of useful applications of “tuning” redox potentials exist (see *e.g.*(9,10)). However, such successes have been based generally on qualitative insight and trial-and-error approaches. Alternative predictive methods for redox properties of proteins, whether designed *de novo* or engineered natural proteins, could significantly accelerate applications in engineering biological systems at the molecular level.

Biomolecular simulations, such as equilibrium molecular dynamics (MD) simulations, can contribute to such developments. Simulations are increasingly assisting the design of proteins, *e.g.*, as catalysts.^11^ Such approaches, which can be used qualitatively to predict the stability of designs, are becoming increasingly capable of predicting thermodynamic properties. Nonetheless, reliably estimating the redox properties of proteins using theoretical-computational approaches remains challenging.^12−20^ Challenges include, for example, a proper dynamic representation of the different redox states and their electrostatic interactions and sampling the relevant conformational states (*e.g.*^13,15^).

Different types of computational techniques have been used to study redox processes in proteins, including MD simulations and continuum electrostatics (CE) calculations.^12−15^ For example, in MD-based free energy simulations, the protein’s conformation changes are explicitly treated for a fixed reduction/protonation state. However, such calculations are computationally expensive.

CE methods have also been widely used to predict changes in protonation and reduction in proteins (*e.g.*^21−23^). These methods are much faster than MD-based approaches, as they sacrifice configuration aspects of the protein and/or solvent. Nonetheless, for most proteins, the lack of explicit dynamics can affect the accuracy of the predictions (*e.g.*^15^). Hybrid approaches, combining MD and continuum electrostatics-based methods, have also been developed to estimate protonation and reduction changes.^24,25^ Such methods require adequate sampling of conformational space, which is still a challenge for most proteins.^21^

Meanwhile, the emergence of stochastic thermodynamics has introduced detailed and integral fluctuation relations that beautifully capture the properties of a wide variety of nonequilibrium processes.^26−28^ They link the distributions, *p*(·), and averages, ⟨·⟩, of stochastically fluctuating quantities, such as entropy *S*, work *W*, or heat *Q*, for a particular process Λ with those of the time-reversed process . Fluctuation relations establish limits on the microscopic fluctuations of small systems that are much more detailed than the macroscopic laws of thermodynamics.^29,30^ Practically, they are used to infer free energy differences Δ*G* based on data from highly nonequilibrium experiments. For example, a range of optical tweezer experiments have been conducted that mechanically stretch single molecules under a variety of conditions and collect the nonequilibrium statistics.^31−33^ Combined with fluctuation relations, these experiments have been used, for example, to determine ligand binding energies as well as characterize the selectivity and allosteric effects of nucleic acids and peptides.^33^

**Figure 1:**
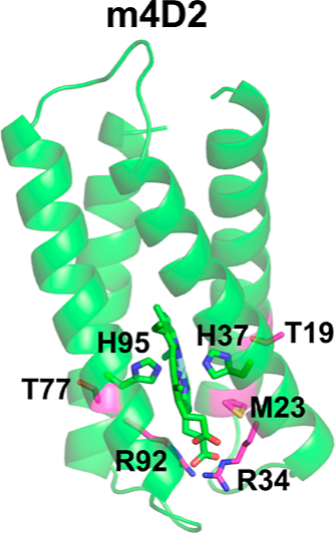
Predicted structure of the *de novo* monoheme-binding maquette protein m4D2. The structure shown was built using Rosetta^37^ (for more details, see^38^). m4D2 is a designed, *de novo* four-helical bundle protein that binds heme B. Heme B and the histidine residues axially coordinating the Fe atom are shown with green sticks, whereas the sites of the mutations studied here are shown with magenta sticks.

Here, we use a detailed fluctuation relation, the Crooks relation,^28^ with data from MD simulations to calculate protein redox potential changes. To the best of our knowledge, this is the first application of these relationships in this context. We use this method to predict the redox potential *E* of a *de novo* designed protein, the m4D2 “maquette” (Figure 1), and several mutants (single and double). m4D2 is a well-characterized soluble four-helix bundle monoheme-binding protein. Redox potentials have been experimentally determined for m4D2 and several mutants by optically transparent thin-layer electrochemistry.^34^ m4D2 is quite small (about 110 residues long), making it an amenable target for MD simulations.^34^ It also lacks some of the complexities of natural proteins, such as allosteric regulation.^34−36^

## 2 Materials and Methods

### 2.1 m4D2 Structure

The computational design of the monoheme m4D2 structure was performed using Rosetta,^37^ as described in detail in ref (38). In our design (Figure 1), the heme group is coordinated by two histidines situated on diagonally opposed helices and is positioned with its propionate groups pointing toward the end of the bundle. In m4D2, there are two threonine residues (threonines 19 and 77), which are directly involved in key hydrogen bonding interactions with the heme-coordinating histidines. Experimental mutation of threonine 19 to an aspartate^38^ changes the redox potential by −28 mV relative to m4D2 (T19D in Table 1). Replacing both threonine 19 and 77 with aspartate^38^ has a substantial effect on the heme redox potential, decreasing it by −56 mV relative to m4D2 (see double mutant, DM, in Table 1). These residues were originally selected for mutation as there is an aspartate in an equivalent histidine-interacting position in horseradish peroxidase, which is believed to play a critical role in the enzyme’s catalytic triad by increasing the imidazolate character of the proximal histidine.^39,40^ This local increase of the negative charge was expected to lower the redox potential of the heme.

**Table 1:** Experimental Redox Potentials *E* (Column 2) and Corresponding Changes δ*E* Relative to m4D2 (Column 3), Where the Double Mutant (DM) is T19D-T77D^a^

protein	experiment (mV)		predicted δ*E* (mV)	
	*E*	δ*E*	MD + CB	PB + MC
m4D2^38^	–118 (1)	0	0	0
T19D^38^	–146 (1)	–28 (1)	–4 (2)	–35
M23N	–119 (1)	1 (1)	14 (2)	0
R34Q	–149 (1)	–31 (1)	–12 (2)	–11
R92Q	–150 (1)	–32 (1)	–14 (2)	–14
DM^38^	–174 (1)	–56 (1)	–12 (2)	–67

a Previously measured redox potentials^38^ are indicated in the table. Calculated redox potential changes relative to m4D2 using the proposed MD + CB method, which postprocesses the data generated by the MD simulations *via* the Crooks-Bayes estimator (3), are shown in column 4. Calculated redox potential changes relative to m4D2 using a well-established CE approach (column 5), combining PB calculations and MC simulations (PB + MC).^23,74^ The errors associated with δ*E* were propagated from those for *E*.

Arginines 34 and 92 are located on the second and fourth helices of m4D2, respectively, and likely interact directly with the heme propionates through ion pairing and hydrogen bonding, as observed in the 4D2 crystal structure.^38,41^ The distance between these arginines and the propionate groups was monitored in our trajectories (Figure S9 in the Supporting Information), and as predicted, these residues can form direct interactions with the negatively charged propionates. Mutation of either of these arginines is likely to alter the network of interactions involving propionate groups. Experimentally, replacement of either arginines by glutamine (so removing the positive charge) is here found to change the redox potential by approximately −30 mV (see R34Q and R92Q in Table 1).

Finally, methionine 23, which is located on helix 1, is also close to the heme. Nonetheless, despite its proximity to the heme, experimentally mutating this methionine to asparagine (see M23N in Table 1) has little effect on the redox potential of the heme (with a +1 mV change relative to m4D2).^38^

### 2.2 MD Simulations

MD simulations were used to generate ensembles of conformations of m4D2,^41^ four single mutants (T19D, M23N, R34Q, and R92Q) and a double mutant, namely, T19D-T77D (hereafter labeled DM). The m4D2 model^38^ produced by Rosetta, described above in Section 2.1, was used as the starting point for the m4D2 simulations. Starting structures for simulations of the mutants were created using the mutagenesis tool in PyMOL.^42^ For each protein, MD simulations were performed for the reduced and oxidized forms.

MD simulations were performed using GROMACS^43−46^ on the University of Bristol’s compute cluster, BluePebble. The GROMOS 54A7 force field^47^ was used for protein, and parameters for the oxidized and reduced redox centers were taken from our previous work.^38^ Protein models were solvated in dodecahedral boxes, with a minimum distance of 2 nm between the protein and the box limits. The simple point charge (SPC) water model^48^ was used. The total net charges of m4D2 and M23N in the oxidized and reduced states are −2 and −3, respectively. For the T19D, R34Q, and R91Q mutants, the overall charge of the proteins in the oxidized and reduced states is −3 and −4. Finally, the total charges for the double mutant (in which aspartate residues replaced both T19 and T77) are −4 and −5 for the oxidized and reduced states, respectively. The overall net charge in all systems was neutralized by adding the exact number of positively charged ions to offset the net charge on the proteins. Overall, 2 and 3 sodium (Na^+^) ions were added in the oxidized/reduced m4D2 and M23N systems; 3 and 4 Na^+^ ions were included in the T19D, R33Q, and R91Q systems; and 4 and 5 Na^+^ ions were added to the double mutant system. In addition to the ions needed to neutralize the systems (*i.e.*, to make the total net charge of the proteins equal to 0), an ionic concentration of 0.05 M sodium chloride was also included in the simulation boxes to mimic the experimental conditions.

Simulations were performed at constant temperature and pressure using the velocity rescaling thermostat^49^ at *T* = 298 K and the Parrinello–Rahman barostat^50,51^ to maintain the pressure at 1 bar. A time step of 2 fs was used for integrating the equations of motion. The LINCS algorithm^52^ was used to constrain bonds in the protein, and the SETTLE algorithm^53^ was used to keep water molecules rigid. Long-range electrostatic interactions were calculated using the particle mesh Ewald method,^54^ with a Fourier grid spacing of 0.12 nm and a 1.4 nm cutoff for direct contributions. 1000 steps of energy minimization with the steepest descent method with harmonic restraints applied to heavy atoms, followed by a further 1000 steps restraining the Cα atoms only, and then 1000 steps with no restraints, were performed prior to MD simulation. Then, a 3 ns restrained MD relaxation was performed to relax the system prior to unrestrained MD simulations.

Multiple MD simulations were performed for each system and redox state: ten 500 ns unrestrained MD simulations were performed for the reduced and oxidized states of m4D2 and for the single mutants (T19D, M23N, R34Q, and R92Q). Twenty 500 ns unrestrained MD simulations were performed for the T19D-T77D double mutant (DM) in the reduced and oxidized states. The replicas were initiated with different sets of random velocities. In total, this amounts to 70 μs of simulation. All analyses were performed using the GROMACS package^43−46^ and in-house tools. PyMOL^42^ was utilized for molecular representation.

All proteins were stable over the simulation time. The simulations showed that the structures of all of the mutants are overall similar to those of m4D2, as expected. The proteins all appeared to be equilibrated after 100 ns (see Figures S2–S7 in Supporting Information). The first 100 ns were taken as equilibration, and only the last 400 ns were analyzed (Figures S1–S8 in Supporting Information), unless stated otherwise. Principal component analysis was used to evaluate the sampling of the conformational space by the replicates (see Figures S5 and S6). To analyze the dynamical changes caused by the mutations, root-mean-square fluctuation (RMSF) profiles of the Cα atoms were calculated (Figure S8). The RMSF plots show that the effects of the mutations are localized to the regions surrounding the mutation site. Some decrease local fluctuations (*e.g.*, T19D), while others increase them (*e.g.*, R92Q) relative to m4D2. The RMSF profiles also show that the unstructured loop regions of the proteins are very mobile, representing some of the largest peaks observed in Figure S8. Such dynamic behavior is also contributing to the high RMSD values observed in Figure S2 in the Supporting Information. As can be seen in Figures S3 and S4, the RMSD profiles for the structured parts of the protein (*i.e.*, excluding the loop regions) show lower Cα deviations from the structures used as starting points for the simulations. These analyses altogether indicate that the MD simulations provide a reasonable conformational sample for the calculations of redox potentials.

### 2.3 Nonequilibrium Perturbations

To determine the energy cost of reducing and oxidizing the heme group, conformations were extracted every nanosecond from the equilibrated trajectories (400 conformations per replicate) and used as starting points for the reduction/oxidation events (in a total of 4000 conformations per system for m4D2, T19D, M23N, R34Q, and R92Q, and 8000 conformations for the DM). In each of these extracted conformations, the redox state of the heme was (instantaneously) changed.

For each conformation, the energy change associated with the oxidation and reduction of the protein was calculated as the difference in energy between the states in that conformation. Note that these energy differences, which were obtained using a molecular mechanics force field, do not take into account the electronic effects associated with adding/removing an electron from the heme group, *i.e.*, the intrinsic energy for reduction/oxidation of the heme cofactor (the intramolecular contribution to the energy).

The energy difference (Δϵ) between oxidation states in the protein was determined using the Kubo-Onsager approach:^55−58^ specifically, by calculating the difference in the potential energy of the protein between every pair of reduced/oxidized conformations extracted from the simulations. The large number (thousands) of replicates allows for convergence of the calculated energetics associated with heme reduction and oxidation.

Note that in this work, the oxidation/reduction reorganization energies are obtained by determining the instantaneous energy difference between redox states. This is a simple way to determine the work value *W* (for more details see Section 2.4). An interesting future extension would be to use dynamical nonequilibrium molecular dynamics (D-NEMD) simulations^55,56,58^ to estimate such energies. Additional factors (*e.g.*, barostat effects) need to be considered for this. Note also that for the energy difference between the two redox states extracted from the MD simulation, we will include only the contribution from the protein. It is clear that the solvent can make a nontrivial contribution. However, when taking the energy differences between states of the protein plus the full solvent, we found that the fluctuations were then too large in comparison to the redox potential we wanted to extract. A solution to this problem would be to include only a portion of the solvent. But this just shifts the problem of where to make the cut. Future theoretical development is needed to address how to adequately include the solvent effects.

It should be noted that the experimental redox potential shifts, while they might appear large, correspond, in fact, to very small free energies relative to the systematic and statistical errors associated with a typical computational calculation. Indeed, this is why predicting redox potential shifts is such a challenging task. Besides the contribution of the solvent, there are other factors that are not considered and can affect the energy differences. These include quantum effects which are not captured by molecular mechanics approaches, such as changes in the heme’s polarization and density and ionization energy due to different environments. Sampling problems, imprecision in the model produced by Rosetta, biases introduced when building the models for the mutants, force field limitations (*e.g.*, the lack of polarization), and uncertainties in the protonation states of the titratable residues are all examples of factors that can affect the energy differences and, thus, computational predictions.

### 2.4 Fluctuation Relations

The goal of this investigation is to predict redox potentials *E* using the fluctuation relations applied to the data from these perturbations. We employ a Bayesian generalization of the procedure to estimate free energy differences via the Crooks fluctuation relation.^28,59^

First, we introduce the Crooks relation. This detailed fluctuation relation is formulated for a generic system, such as a harmonic oscillator or a molecule, with at least one externally controlled parameter λ with two settings *A* and *B*. For example, the harmonic potential’s frequency can be either λ_A_ or λ_B_, and in a molecule, a charge can be absent (λ_A_) or present (λ_B_). Starting at setting λ_A_, with an equilibrium state at inverse temperature β = 1/*k*_B_*T*, where *k*_B_ is the Boltzmann constant, the system is pushed (arbitrarily far) out of equilibrium by varying the parameter λ with some protocol Λ. This can be either a smooth variation in time, λ(*t*), or an instantaneous change, *e.g.*, λ_A_ → λ_B_.

Work *W* is received by the system during the action of the nonequilibrium protocol. Crooks’ relation^28^ quantifies the likelihood *p* of a specific work value *W* being required given the forward protocol Λ in comparison to the likelihood of the corresponding negative value – *W* being required given the backward protocol , *i.e.*
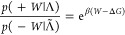
1While the left-hand side contains nonequilibrium work distributions, the right-hand side contains equilibrium properties of the system at settings λ_A_ and λ_B_. Specifically, Δ*G* = *G*_B_ – *G*_A_ is the free energy difference between the two equilibrium states at the inverse temperature β. Obviously, in a quasistatic protocol Λ_qs_ where the system is always in equilibrium, one would have  and *W* = Δ*G* in every run, as expected from macroscopic thermodynamics. The power of relation (1) is that it is valid for protocols that drive the system far from equilibrium.

In the context of our heme-containing proteins, the two settings are that an electron is absent (setting λ_A_, oxidized) or present (setting λ_B_, reduced). The free energy difference for reduction is then Δ*G* = *G*_B_ – *G*_A_ = −*nFE*,^60^ where *E* is the redox potential of the heme, *F* is Faraday’s constant (96485.3 C mol^–1^), and *n* is the number of electrons being transferred. In our case, *n* = 1, so that Δ*G* = −*FE*.

The reduction of the heme group is thus identified with the forward protocol Λ, while the oxidation process is the backward protocol Λ̃. To get the work values for the reduction (oxidation) protocols, we first note that there is no heat contribution since the simulations implement an *instantaneous* appearance (disappearance) of the electron in the heme, leaving no time for heat to be exchanged.^27,30^ We define the statistical work value *W*_*i*_, received by the heme in the *i*-th reduction process Λ as the statistical energy difference Λ̃ for *i* = 1, ..., μ. *I.e.*, in each run, an equilibrium simulation gives the initial statistical energy value of the oxidized protein, ϵ_ox_^*i*^, and a subsequent nonequilibrium perturbation, where the electron has been removed, gives the final statistical energy value, ϵ_red_^*i*^, for the reduced protein. Similarly, for the backward protocol Λ̃, the statistical work is *W*_*i*_ = ϵ_ox_^*i*^ – ϵ_red_^*i*^ = Δϵ_*i*_ for *i* = μ + 1, ..., 2 μ. For an ensemble of nonequilibrium processes, for a given m4D2 mutant, we obtain a set of work values ***W*** = (*W*_1_, ..., *W*_2 μ_), where entries 1, ..., μ correspond to reduction and entries μ + 1, ..., 2 μ to oxidation.

**Figure 2:**
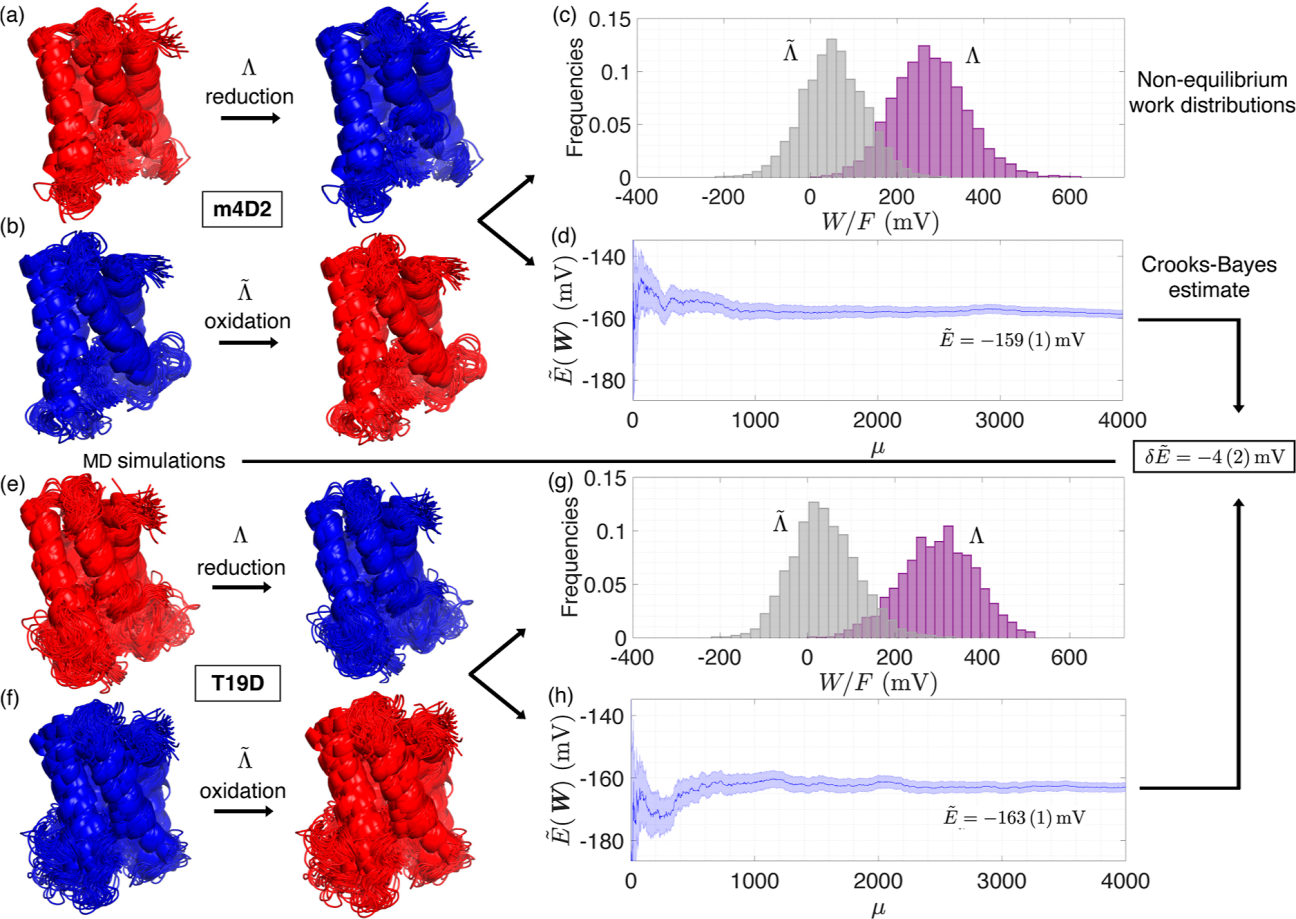
Schematic of the MD + CB method used to calculate the redox potential shift (δ*E*) of T19D relative to m4D2. MD simulations and a nonequilibrium perturbation were used to determine the energy cost of reduction [panels (a,e)] and oxidation [panels (b,f)] for m4D2 and T19D. Reduction and oxidation are identified as the forward (Λ) and backward  protocols, respectively, which are needed as input for the detailed Crooks fluctuation relation. The statistical work, *W*, was determined as *W* = ϵ_fin_ – ϵ_ini_, where for the reduction process, the final energy is the energy for the reduced protein, ϵ_fin_ = ϵ_red_, and the initial energy is the energy for the oxidized protein, ϵ_ini_ = ϵ_ox_. For the oxidation process, it is the other way around, *i.e.*, ϵ_fin_ = ϵ_ox_ and ϵ_ini_ = ϵ_red_. The resulting work histograms, *p*(*W*|Λ) for the forward/reduction (purple) process and  for the backward/oxidation (gray) process, are shown in panels (c) for m4D2 and (g) for T19D. These histograms are shown for illustrative purposes, but they are not employed to perform the estimation of redox potentials due to the finite-sample caveats discussed in the main text. Instead, we use the more accurate Crooks-Bayes estimate^59^ (3), with error (4). For m4D2 and T19D, respectively, panels (d) and (h) show the convergence of these estimates as the number of iterations μ increases. The estimate δ for the shift δ*E* was calculated by subtracting the *E*-estimate  for m4D2 from that for T19D, and the errors were propagated. The same procedure was applied to all of the mutants, with results given in Table 1.

When information from both directions of a process, Λ and Λ̃, is available, the commonly used procedure to estimate Δ*G* is via the Crooks relation (1), as follows:^28,30^ Constructing the forward and backward work histograms, *p*(*W*|Λ) and , one identifies the point W* where they cross, *i.e.*. By virtue of the Crooks relation (1) this gives an estimate^33^ for free energy as Δ*G* = W*. For the m4D2 protein, this is illustrated in Figure 2c, and in Figures 2g and S13 for the mutants. It should be noted that the work distributions in Figures 2c,g and S13, corresponding to the nonequilibrium work values associated with instantaneous oxidation/reduction processes, can also be viewed as equilibrium distributions obtained from sampling the oxidized and reduced states.

However, this histogram-based method has the caveat that it requires a sufficiently large number of iterations to produce good estimates.^61^ Not only are finite statistics known to significantly impact the quality of standard free energy estimates^62−64^ but using estimators whose validity depends on the size of the sample poses the risk of amplifying potential errors due to limited sampling in MD simulations. To address these caveats, Maragakis *et al.*(59) put forward a *Bayesian* framework for the estimation of free energy differences that combines the Crooks relation with the Bayes theorem.^61,65^ The key advantage of using the Bayes theorem is that it can extract all the information available in a given sample, regardless of its size.^59,61,66^ Therefore, it can help to prevent the amplification of limited sampling.

To use this approach, one first calculates the conditional probability density *p*(Δ*g*|***W***), where Δ*g* denotes a hypothesis about the true free energy difference Δ*G* given the work values ***W*** provided by the MD simulations. Following Maragakis *et al.*,^59^ we use
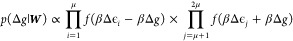
2where *f*(*x*) = 1/[1 + exp(−*x*)] is the logistic function, and β = 1/(*k*_B_*T*) is the inverse temperature used in the simulations (*i.e.*, *T* = 298 K). Equation 2 is derived using minimal prior information; see details in the Supporting Information subsection S.3.

The probability distribution *p*(Δ*g*|***W***), shown in Figure S14 for m4D2 and its mutants, contains all the information available to estimate Δ*G*. To map this information into a concrete value for the redox potential *E*, one uses the estimator (indicated by a tilde)
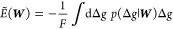
3where *F* is the Faraday constant. This estimator is optimal under the square error criterion,^59,61^ with error
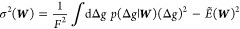
4

The use of energy differences Δϵ obtained from MD simulations of proteins (see Section 2.3), together with the Crooks-Bayes estimator given in eq 3 for probability distribution (2), constitutes a new computational method for the prediction of redox potentials. We refer to this method as the MD + CB method; see illustration in Figure 2. The redox potential shifts of the mutants relative to m4D2 obtained with the MD + CB method are reported in Table 1.

### 2.5 Note on Other Methods

Given the MD simulated work values, a variety of estimation methods exist to infer the free energy difference Δ*G*. One of them is using Bennett’s acceptance ratio (BAR).^67,68^ The BAR estimation method has been shown to be akin to combining Crooks’ relation with maximum likelihood estimation.^59,69^ This makes its reliability generally justified only in the limit of an asymptotically large number of work measurements.^70^ In contrast, the Crooks-Bayes approach we use here leads to reliable estimates, even for small data sets. This is a general property of Bayesian estimation techniques, enabled by the inclusion of prior information (or the absence of it), such as symmetries,^71^ in the calculations of estimators and errors.^61,65^

Another common method is using Jarzynski’s equality,^27^ ⟨exp(−β*W*)⟩ = exp(−βΔ*G*), an integral fluctuation relation that can be deduced from the Crooks relation. Jarzynski’s equality provides a useful estimator for Δ*G* for experiments conducted out of equilibrium that can only implement one direction of the protocol.^31,72^ It is necessarily less informative than using the Crooks relation because the average over a work probability density is less informative than the probability density itself. Note that analogous considerations apply to any other method based on work averages for one direction of the protocol. This includes, *e.g.*, Zwanzig’s free energy perturbation (FEP) formula,^73^ which coincides with Jarzynski’s estimator for instantaneous changes, and the linear response (LR) approximation, which is based on the assumption that work distributions are Gaussian.

Relationships between various free energy estimation methods based on work averages or distributions have been discussed in the literature.^27,59,69,73^ Hence, in addition to comparing the predictions of the new MD + CB method to experimental results, we chose to compare them to the predictions of a method that is completely different. This approach, which we abbreviate PB + MC, is based on the widely used continuum electrostatic method^23,74^ and is known to perform reasonably well for these systems, providing a baseline for practical protein engineering applications.

The change in redox potential of the heme group between m4D2 and mutants has previously been calculated with this approach.^23,74^ This method involves simulating the joint binding equilibrium of the proton and electrons. It uses a combination of Poisson–Boltzmann (PB) calculations, *e.g.*, with MEAD (version2.2.9),^75−77^ and Metropolis Monte Carlo (MC) calculations, using the software PETIT (version 1.6).^78^ The PB calculations compute the individual and pairwise terms needed to obtain the free energies of protonation/reduction changes. These energies are then used in the MC calculations. The changes in the redox potential of the heme group relative to m4D2 are determined from the corresponding reduction curve by extracting the *E*-shift values corresponding to a reduced fraction of 0.5 in Figure S11.

The structural model predicted by Rosetta^38^ was used for the calculations of m4D2, while models for the mutants were constructed using PyMOL.^42^ One structure for each system, namely, m4D2, T19D, M23N, R34Q, R92Q, and the DM, was used for the calculation. We note that a slightly different structural preparation protocol prior to the calculations gives slightly different results.^38^ The charges for all the atoms in the protein (except the heme group) and radii were taken from the GROMOS 54A7 force field^47^ using a previously described procedure.^79^ The partial charges for the heme group were taken from our previous work.^38^ These calculations use a temperature of 298 K and a molecular surface defined by a solvent probe radius of 1.4 Å.^79^ The dielectric constants used for the solvent (ε_sol_) and for the protein (ε_protein_) were 80 and 20, respectively.^79^ An ionic strength of 0.05 M was used. The finite-difference linear PB calculations used a three-step focusing procedure^80^ employing consecutive grid spacings of 1.0, 0.5, and 0.25°A. Each MC calculation comprised 10^5^ MC steps, and the acceptance/rejection of each step followed a Metropolis criterion^81^ using the PB free energies.

We refer to this alternative method as the PB + MC method and report the predicted redox shifts of the m4D2 mutants in column 5 in Table 1.

### 2.6 Experimental Redox Potentials

The heme reduction potentials of the M23N, R34Q, and R92Q variants were determined here (Figure S16) using optically transparent thin-layer electrochemistry^82^ using methods previously used for m4D2 and other mutants.^38^ 120 μL of *de novo* protein samples were mixed with 12 μL of glycerol and 0.5 μL each of indigotrisulfonic acid, 2-hydroxy-1,4-naphthoquinone, phenazine, anthroquinone-2-sulfonate, and benzyl viologen mediators at approximately 10 μM concentration. The mediators are used to facilitate electron transfer between the working electrode and the heme cofactor and therefore promote rapid equilibration in the electrochemical cell.^83^

To obtain the reduction potentials of the proteins, a Biologic SP-150 was used to apply stepwise potentials between a thin platinum gauze working electrode and a platinum counter electrode, typically over a range of −525 to –225 mV *vs* a Ag/AgCl reference electrode also in the electrochemical cell. The protein sample and thin Pt gauze working electrode were housed in a modified quartz EPR cuvette (Wilmad), with a path length of 0.3 mm, and the counter and reference electrodes were held above in a glycerol-free buffer layer within a fused glass side arm tube. UV–visible absorbance spectra were recorded between 200 and 800 nm after 30 min of equilibration at each potential to measure the evolution of the heme absorbance spectrum as cycled between ferric and ferrous states during reductive and oxidative sweeps of potential. Redox potential measurements of horse heart cyctochrome *c* and m4D2 were used to calibrate the Ag/AgCl reference electrode for each round of redox measurements, enabling reduction potentials to be quoted *versus* the Nernst hydrogen electrode (NHE). The experiments were conducted at room temperature (*ca. T* = 298 K).

For these *b*-type heme proteins with bis-histidine coordination, *A*_416nm_ represents the position of the oxidized ferric Soret band, and *A*_429nm_ represents the position of the reduced, ferrous Soret band. The Δ*A*_429nm_ was plotted against the applied potential (mV), and the Nernst equation was used to calculate the redox midpoint potential (*E*)
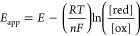
5where *E*_app_ and *E* are the applied potential and the redox potential, respectively; *R* is the universal gas constant; *T* is the temperature; *n* is the number of electrons being transferred; [red] is the concentration of the reduced ferrous heme; [ox] is the concentration of the oxidized ferric heme; and *F* is Faraday’s constant. For a normal reduction reaction such as
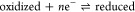
6the general Nernst eq 5 can be used to describe the redox potential under nonstandard conditions, such as those in which the data were collected.

For these experiments, data was collected in triplicate and then processed using a Jupyter Notebook. Normalized mean data was fit to a 1-electron Nernst equation using the SciPy optimize curve_fit function.^84^

## 3 Results and Discussion

Multiple long MD simulations were performed for the oxidized and reduced states of m4D2, four single mutants (T19D, M23N, R34Q, and R92Q), and the T19D-T77D double mutant (DM). The trajectories provided ensembles of conformations (4000 for m4D2 and single mutants and 8000 for the double mutant) to calculate the instantaneous oxidation and reduction processes of the proteins, as described in Section 2.3, using the approach illustrated in Figure 2.

As outlined in Section 2.4, fluctuation relations were then used to calculate redox potential shifts for the mutants relative to m4D2 (see results in Table 1), using the energy differences described above. The energy changes between the reduced and oxidized states were used to determine the statistical work values associated with reduction and oxidation for each of the six proteins. These work values (Figure S12) were used to calculate the probability density (2). This encodes the information that the MD simulations provide about the redox potential according to the Crooks relation. All six densities are reported in full in Figure S14. These probabilities were used to calculate the Crooks-Bayes estimates (eq 3). The redox potential changes calculated in this way are given (relative to m4D2) in Table 1 (column 4). The uncertainties for these values were propagated from the mean square errors (4) for each redox potential and are indicated in brackets. The results for all six proteins showed statistical convergence from μ ≃ 2000 data points (Figure S15).

While eq 3 is in principle capable of predicting absolute redox potentials (see Table S1 in the Supporting Information), it should be noted that the energy differences from the simulations used here do not account for quantum effects (such as polarization and ionization energy), as those are not captured by molecular mechanics approaches. Therefore, we report the redox potentials as changes relative to m4D2, denoted as δ*E*; see Table 1.

The calculated redox potential shifts for the m4D2 mutants determined using the Crooks relation here generally correlate well with the experimental values Table 1 (columns 3, 4). For T19D, the change in redox potential predicted by the MD + CB result is −4 mV (experimental shift is −28 mV), whereas for M23N it is 14 mV (experimental shift is +1 mV). The MD + CB predictions for the two arginine-to-glutamine mutants, namely, R34Q and R92Q, are very similar (−12 mV for R34Q and −14 mV for R92Q), as also observed experimentally (experimental shifts are −31 and −32 mV for R34Q and R92Q, respectively). The MD + CB predictions show a relatively small shift (−12 mV) for the double mutant, which in experiments displays a large shift of −56 mV. The MD + CB calculated value for the double mutant is notably less negative than the experimental result. Possible reasons for the differences observed between the MD + CB predicted and the experimental redox shifts are discussed below.

The MD + CB calculations correctly predict the sign of the redox potential change for all of the mutants. They also give the correct order of the redox potential shifts for all the single mutants simulated: M23*N* > T19D > R34Q ≈ R92Q. Indeed, for single mutants, the Pearson correlation coefficient is ρ_corr_ = 0.97. Interestingly, for the single mutants, the MD + CB method predicts redox potential shifts that are consistently offset in comparison to the experiment by around −18 mV.

These findings indicate that this approach may be useful for predicting redox potential changes for single mutants. The performance of the MD + CB method is, overall, comparable to that of the PB + MC approach for these proteins.

The good agreement for the single mutants indicates that the MD + CB method can give good results. The discrepancy for the double mutant probably arises from issues of modeling the structure, protonation states, and sampling the conformational landscapes of this mutant in one or both redox states. For the MD + CB method to give good results, it is essential that the MD simulations sample the conformations of each state adequately (giving a representative ensemble of structures for each) and that they overlap sufficiently. Although several microseconds of simulation were performed for each system, the sampling gathered may not be enough to explore the conformations of these mutants in one or both redox states. This is suggested by simulations of, for example, the oxidized state of R92Q, in which we find an unusually persistent direct hydrogen bond between glutamine in position 92 and the heme propionates, present in more than 35% of the total simulation time (Figure S9B). The uncommonly high frequency of this interaction in the oxidized R92Q system suggests that these simulations may be trapped at an energy minimum. This persistent hydrogen bond is indeed observed in five of the ten R92Q trajectories for the oxidized state. Predictions for double mutants are generally more difficult because the structural changes induced by two mutations are generally significantly larger than those for single mutations. The dynamics of the specific double mutant here (T19D–T77D) are significantly altered from those of m4D2 due to the two extra negative charges. The introduced aspartate residues form strong electrostatic interactions with nearby positively charged residues (K36 and K94) (Figure S10). These new salt bridges significantly affect the overall dynamics of the protein, rigidifying it (Figure S8). This may mean that longer simulations or more repeats are required to sample the conformational space of this mutant properly. Overall (and despite the limitations discussed in Section 2.3), our results clearly show that using fluctuation relations to postprocess MD simulation data is a reasonably reliable approach to predicting redox shifts in proteins, given sufficient sampling of both redox states in MD.

To benchmark the MD + CB method, we also calculated the changes in redox potential relative to m4D2 using a well-established CE approach, which combines Poisson–Boltzmann (PB) electrostatic calculations with Metropolis Monte Carlo (MC) simulations.^23,74^ This method, which uses simplified models for both the solvent (dielectric continuum) and the protein (atomic point charges immersed in a low dielectric medium), allows fast calculation of the free-energy terms associated with redox and protonation changes.^23,74^ These energy terms are then used to sample protonation and redox states using MC.^23,74^ This PB + MC method has already proven to be a valuable tool for the redox engineering of m4D2: it correctly predicted the order of reduction of the designed m4D2 mutants using predicted structures from Rosetta.^38^

The PB + MC results are presented in (Table 1, column 5) and (Figure S11). Overall, we observe that for some mutants, *e.g.*, T19D and M23N, the PB + MC predictions are closer to the experimentally measured redox shifts, while for others, *e.g.*, R34Q and R92Q, the new method combining MD simulations and fluctuations relations (MD + CB) performs as well as the PB + MC approach.

It should be noted that in the PB + MC calculations, the dynamic behavior of the proteins is implicitly modeled using a dielectric constant. In contrast, a large set of conformations (4000 for m4D2, T19D, M23N, R34Q, and R92Q, and 8000 conformations for the DM) was used for the MD + CB predictions, thus meaning that in this approach, protein dynamics is being explicitly factored into the calculations. This may be an advantage for systems that undergo conformational changes during the reduction/oxidation process. A potentially significant effect included in PB + MC and not in MD + CB is the inclusion of protonation state changes in the protein associated with redox changes. This may account for the better performance of PB + MC in some cases. A potentially useful extension to the MD + CB method would be the inclusion of protonation state changes, *e.g.*, through MC calculations or constant pH MD.

For all the mutants studied here, both methods give results with a similar correlation strength with the experimental values, namely, 0.85 and 0.84 for the MD + CB and PB + MC methods, respectively. However, for the single mutants only, the MD + CB method shows a correlation of 0.97 with the experimental data compared with 0.61 obtained for the PB + MC method—thus reinforcing the earlier claim that fluctuation relations are, when combined with MD simulations, a valid predictive tool for calculating redox potential changes.

## 4 Conclusions

Natural and designed redox proteins are increasingly widely used in technological applications (*e.g.*, biocatalysis and biomolecular electronics). For such applications, there is a need to be able to predict protein redox potentials, *e.g.*, to aid in designing mutations to change the redox potential to optimize it for a particular application. Molecular simulation tools are potentially useful in this context to suggest candidates for experimental characterization and to understand the causes of observed redox potential changes. Here, we have proposed and tested a method for the calculation of redox potentials from MD simulations with the application of fluctuation relations and Bayesian inference. A comparison of the predictions of the MD + CB approach against experimentally measured redox potentials for point variants of a *de novo* heme protein indicates that the method is usefully predictive for relative potential shifts. Comparison with the completely different PB + MC method indicates that the approach proposed here performs similarly well for single-point mutations. The MD + CB method can be readily applied as a complement to standard equilibrium MD simulations of different redox states and so may be useful in protein design and engineering applications.

## Supporting Information Available

Several plots evidencing the conformational stability of the MD simulations; reduction curves for the mutants relative to m4D2 determined using the PB + MC method; mathematical procedure to estimate free energy differences by combining the Crooks fluctuation relation with the Bayes theorem; nonequilibrium work values computed from the simulation data; work histograms for the forward and backward protocols; final posterior probabilities; convergence of our redox potential estimator; experimentally determined redox potentials for the M23N, R34Q, and R92Q mutants; and experimental and predicted redox potentials for m4D2, T19D, M23N, R34Q, R92Q, and T19D-T77D (PDF)

## Notes

The files containing the energy differences extracted from the MD simulations for m4D2 and mutants, as well as the scripts needed for the prediction of the redox potential shifts using the MD + CB approach, are available on GitHub (github.com/jesus-rubiojimenez/FlucRels-CrooksBayes).
