# Supplementary material for: Fluctuation Relations to Calculate Protein Redox Potentials from Molecular Dynamics Simulations
Source: J Chem Theory Comput. 2023 Dec 27;20(1):385–95. doi: 10.1021/acs.jctc.3c00785 (PMC10782445; doi:10.1021/acs.jctc.3c00785)
Supplement: Supplementary file 1 — ct3c00785_si_001.pdf [file ct3c00785_si_001.pdf]

# – Supporting information –

Fluctuation relations to calculate protein redox potentials from molecular dynamics simulations

A. S. F. Oliveira,[†,1,2,3] J. Rubio,[†,4,5] C. E. M. Noble,[2,3] J. L. R. Anderson,[2,3] J. Anders,[*,5,6] and A. J. Mulholland[*,1]

[†] *These authors contributed equally.*

[1] *Centre for Computational Chemistry, School of Chemistry, University of Bristol, Bristol BS8 1TS, UK*
[2] *School of Biochemistry, University of Bristol, Bristol BS8 1DT, UK*
[3] *BrisSynBio Synthetic Biology Research Centre, University of Bristol, Bristol BS8 1TQ, UK*
[4] *School of Mathematics and Physics, University of Surrey, Guildford GU2 7XH, UK*
[5] *Department of Physics and Astronomy, University of Exeter, Stocker Road, Exeter EX4 4QL, UK*
[6] *Institute of Physics and Astronomy, University of Potsdam, 14476 Potsdam, Germany*

* E-mail: Adrian.Mulholland@bristol.ac.uk; janet@qipc.org

In section S.1 below, we display a range of figures to evidence the conformational stability of the MD simulations:

Fig. S1: Average structures of oxidized and reduced m4D2 and its mutants.
Fig. S2: Time evolution of the average Cα RMSD for m4D2 and mutants showing that all systems are equilibrated after 100 ns.
Fig. S3: Time evolution of the average Cα RMSD for all the Cα atoms and for helices 1-4 in the oxidized systems.
Fig. S4: Time evolution of the average Cα RMSD for all the Cα atoms and for helices 1-4 in the reduced systems.
Fig. S5: PCA for m4D2 and mutants showing that the replicates sample different regions of the conformational landscape.
Fig. S6: PCA for the oxidized and reduced m4D2 and mutants showing that the two states sample different regions of conformational space.
Fig. S7: Time evolution of the number of residues without secondary structure showing that the protein structures were stable during the simulation time.
Fig. S8: Average fluctuations for the Cα atoms in m4D2 and mutants showing that in general the mutations affect the dynamics of their surrounding regions.
Fig. S9: Histogram of the distance between residues in position 34 and 92 and the heme propionates for m4D2 and its mutants.
Fig. S10: Histogram of the distance between residue in position 19 and 36, and 77 and 94 for m4D2, T19D and T19D-T77D.

In section S.2 below, we display the following figure:

Fig. S11: Reduction curves for T19D, M23N, R34Q, R92Q and T19D-T77D relative to m4D2 highlighting the mutant's E shift relative to m4D2.

In section S.3 below, we discuss the mathematical procedure to estimate free energy differences by combining the Crooks fluctuation relation with Bayes theorem.

In section S.4 below, we display the following figures:

Fig. S12: Non-equilibrium work values computed from the MD simulation data for m4D2 and its mutants.
Fig. S13: Work histograms for forward and backward protocols obtained from the data in Fig. S12.
Fig. S14: Final posterior probabilities obtained from the data in Fig. S12.
Fig. S15: Convergence of our redox potential estimator $\tilde{E}$ towards the values given in the main text as the number of input data μ increases. Such values are based on the final posterior probabilities shown in Fig. S14.

In section S.5 below, we display the following figure:

Fig. S16: Experimentally determined redox potentials for the M23N, R34Q and R92Q mutants of m4D2.

In section S.6 below, we provide the following table:

Tab. S1: Experimental and predicted redox potentials E for m4D2, T19D, M23N, R34Q, R92Q, and T19D-T77D.

S.1 Conformational stability of the MD simulations

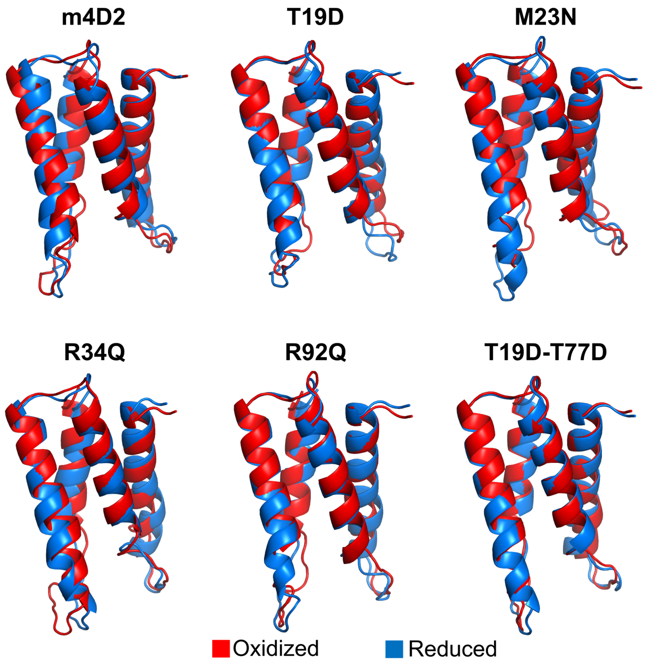

Figure S1: Average structures for the oxidized and reduced m4D2, T19D, M23N, R34Q, R92Q and T19D-T77D. The average structures were calculated using the last 400 ns of all replicates for each system (10 replicates for m4D2 and single mutants and 20 replicates for the double mutant).

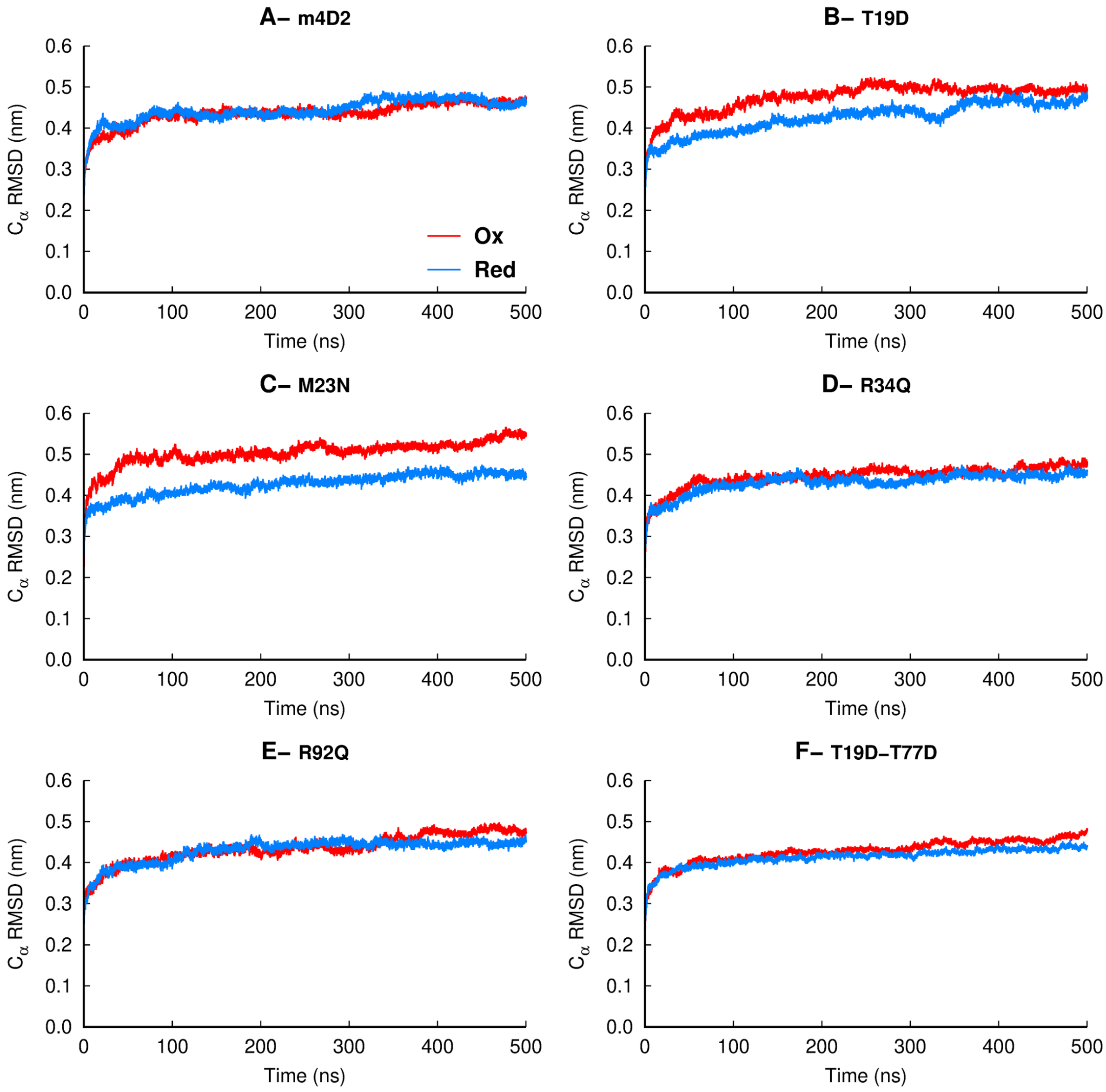

Figure S2: Time evolution of the average Cα RMSD for m4D2 (**A**), T19D (**B**), M23N (**C**), R34Q (**D**), R92Q (**E**) and T19D-T77D (**F**). The Cα RMSD was calculated relative to the starting structures and averaged over all replicates (10 replicates for m4D2 and single mutants and 20 replicates for the double mutant). All the systems were considered equilibrated after 100 ns.

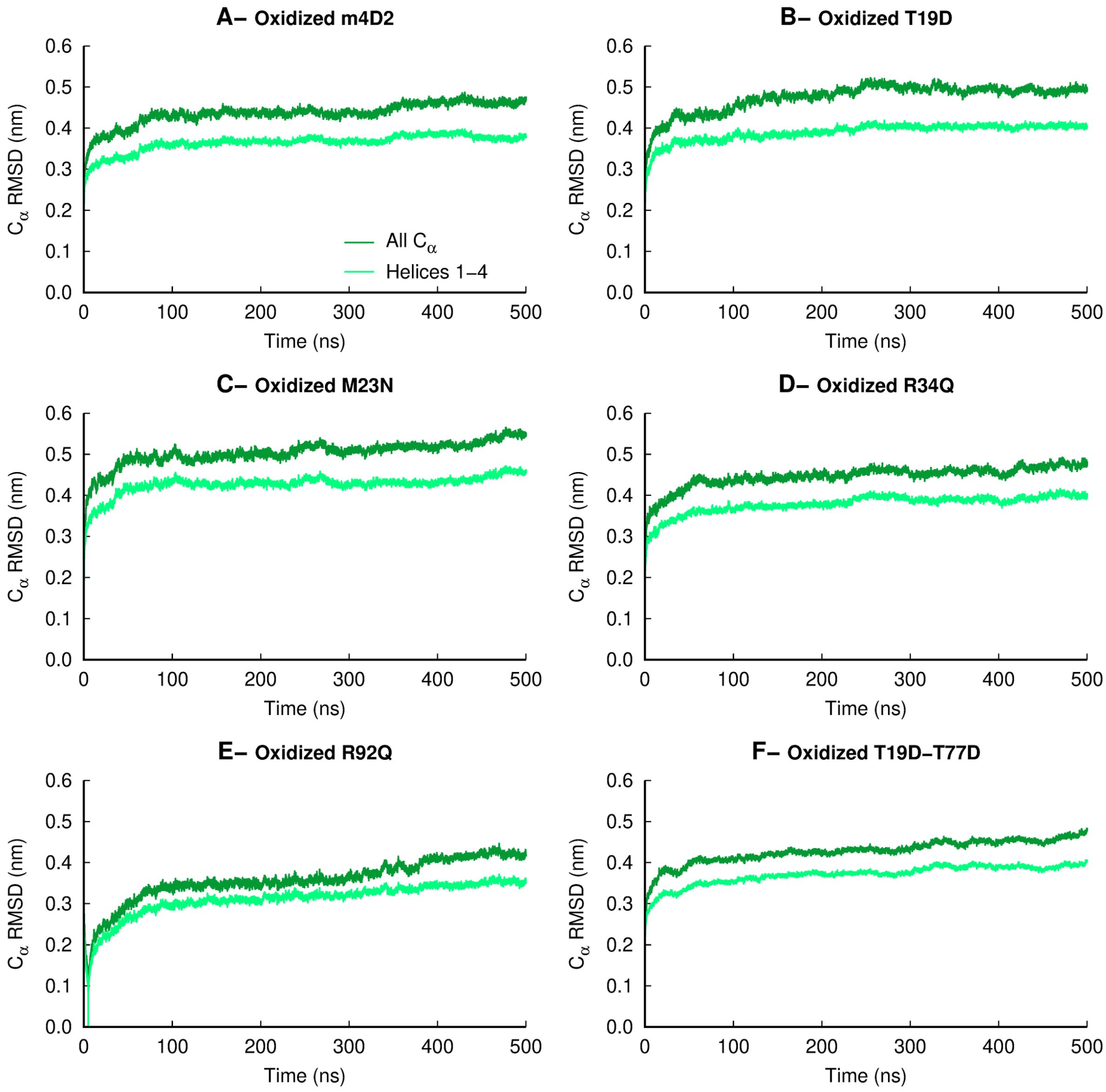

Figure S3: Time evolution of the average Cα RMSD for oxidized m4D2 (**A**), T19D (**B**), M23N (**C**), R34Q (**D**), R92Q (**E**) and T19D-T77D (**F**) relative to the starting structures for all Cα atoms (dark green line) and for the structured regions (light green line) of the proteins. The Cα RMSD was calculated relative to the starting structures and averaged over all replicates (10 replicates for m4D2 and single mutants and 20 replicates for the double mutant). The structured region includes α-helices 1-4. Please note that helices 2 and 4 contain the two histidine residues axially coordinating the heme Fe atom.

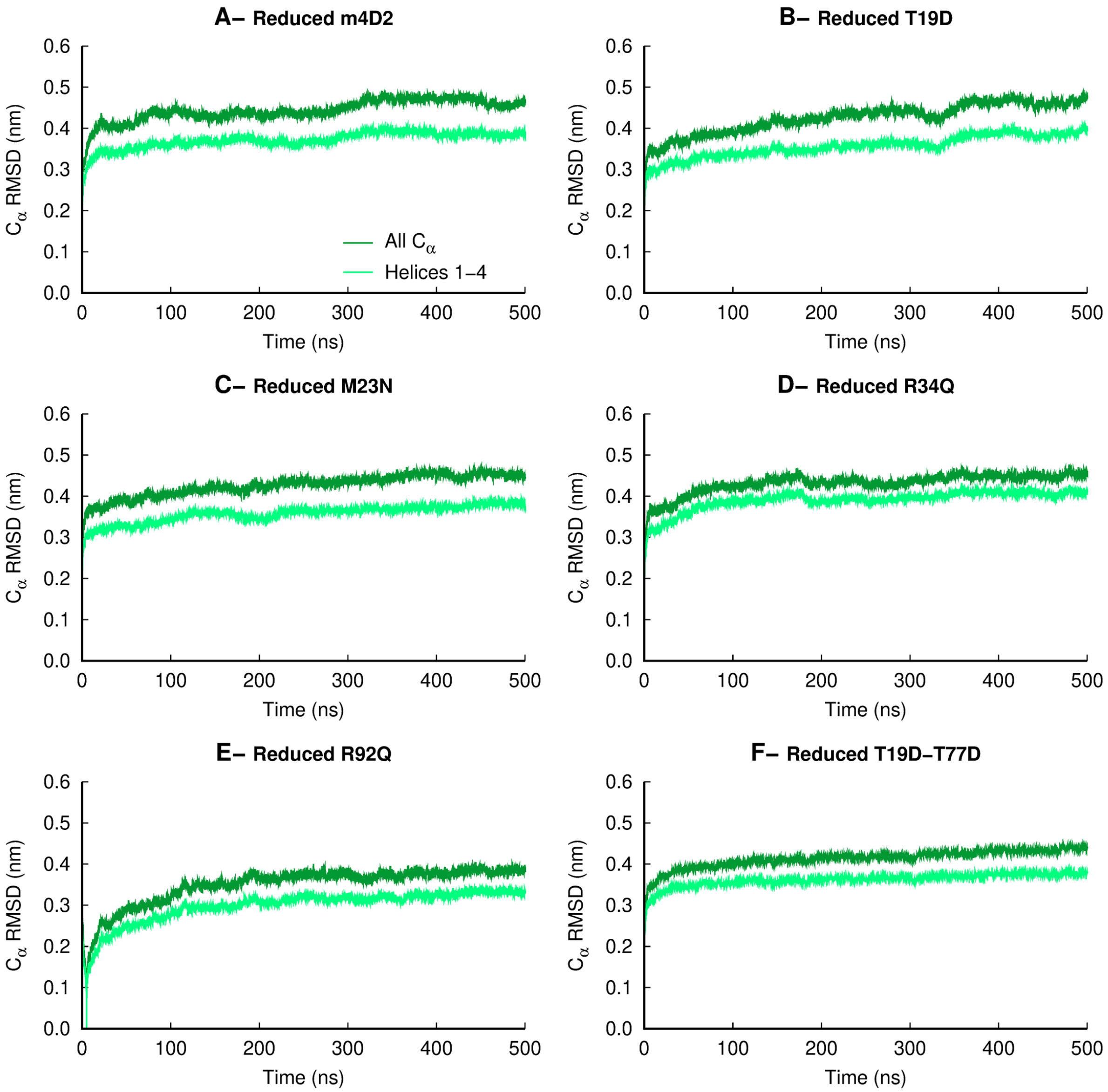

Figure S4: Time evolution of the average Cα RMSD for reduced m4D2 (**A**), T19D (**B**), M23N (**C**), R34Q (**D**), R92Q (**E**) and T19D-T77D (**F**) relative to the starting structures for all Cα atoms (dark green line) and for the structured regions (light green line) of the proteins. The Cα RMSD was calculated relative to the starting structures and averaged over all replicates (10 replicates for m4D2 and single mutants and 20 replicates for the double mutant). The structured region includes α-helices 1-4. Please note that helices 2 and 4 contain the two histidine residues axially coordinating the heme Fe atom.

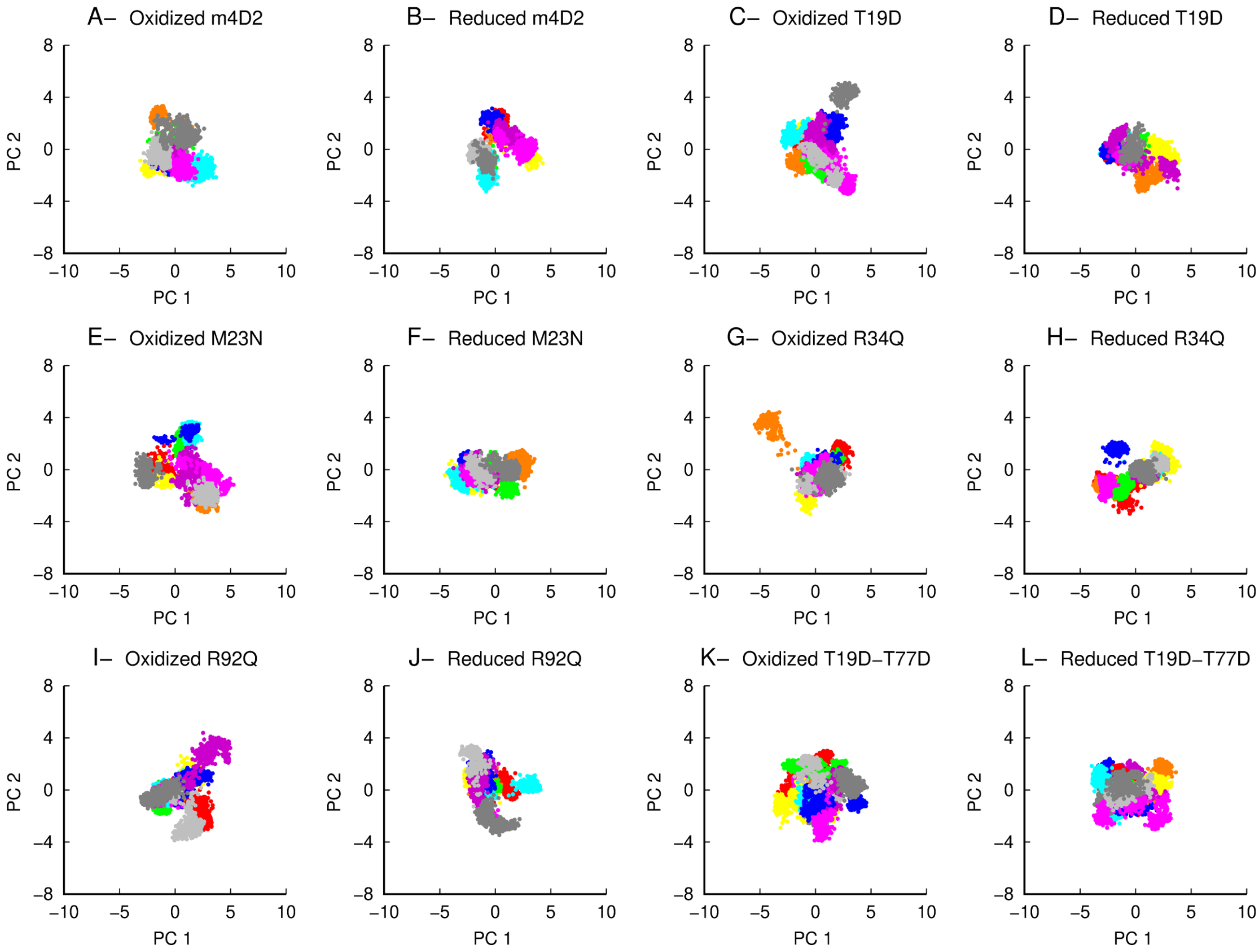

Figure S5: PCA of all replicates for the oxidized and reduced m4D2 (**A-B**), T19D (**C-D**), M23N (**E-F**), R34Q (**G-H**), R92Q (**I-J**) and T19D-T77D (**K-L**). All oxidized and reduced replicates for each system were combined before the analysis, and each trajectory contained one conformation per nanosecond per replicate (totaling 10001 frames for the m4D2 and single mutants and 20001 frames for the double mutant) with all the Cα atoms of the protein. Please zoom into the image for detailed visualization. Note that different replicates sample different regions of conformational space, thus improving the overall sampling for each system.

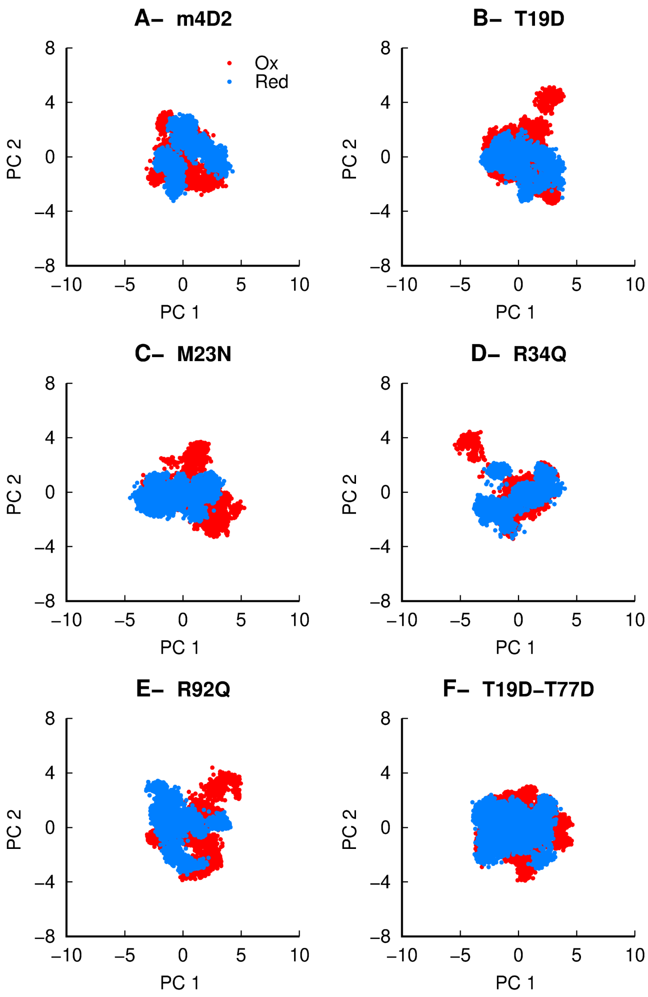

Figure S6: PCA for the m4D2 (**A**), T19D (**B**), M23N (**C**), R34Q (**D**), R92Q (**E**) and T19D-T77D (**F**). All oxidized and reduced replicates for each system were combined before the analysis, and each trajectory contained one conformation per nanosecond per replicate (totaling 10001 frames for the m4D2 and single mutants and 20001 frames for the double mutant) with all the Cα atoms of the protein. Note that the oxidized and reduced systems sample different regions of conformational space.

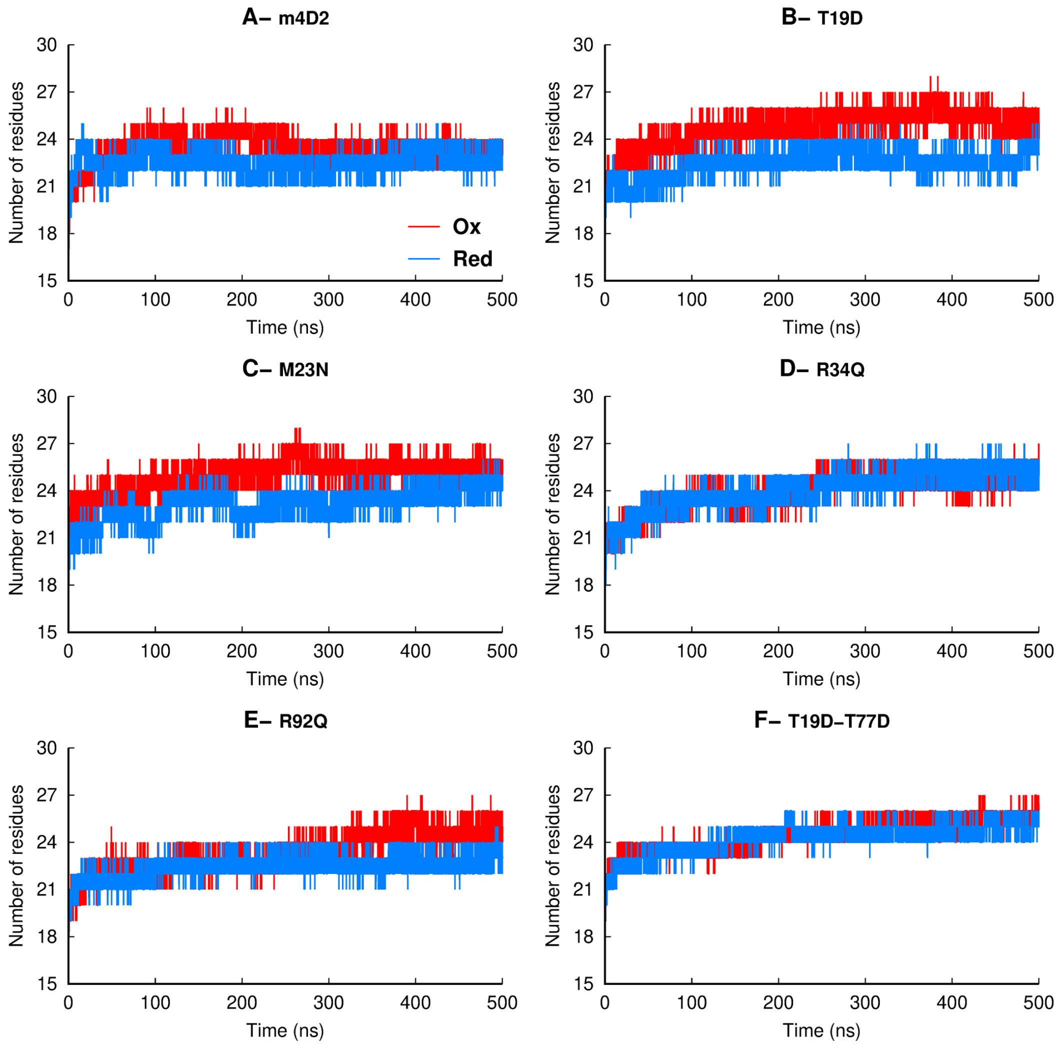

Figure S7: Number of residues with coil secondary structure along the simulation time for the m4D2 (**A**), T19D (**B**), M23N (**C**), R34Q (**D**), R92Q (**E**) and T19D-T77D (**F**). The secondary structure assignment was performed with the DSSP[1] software.

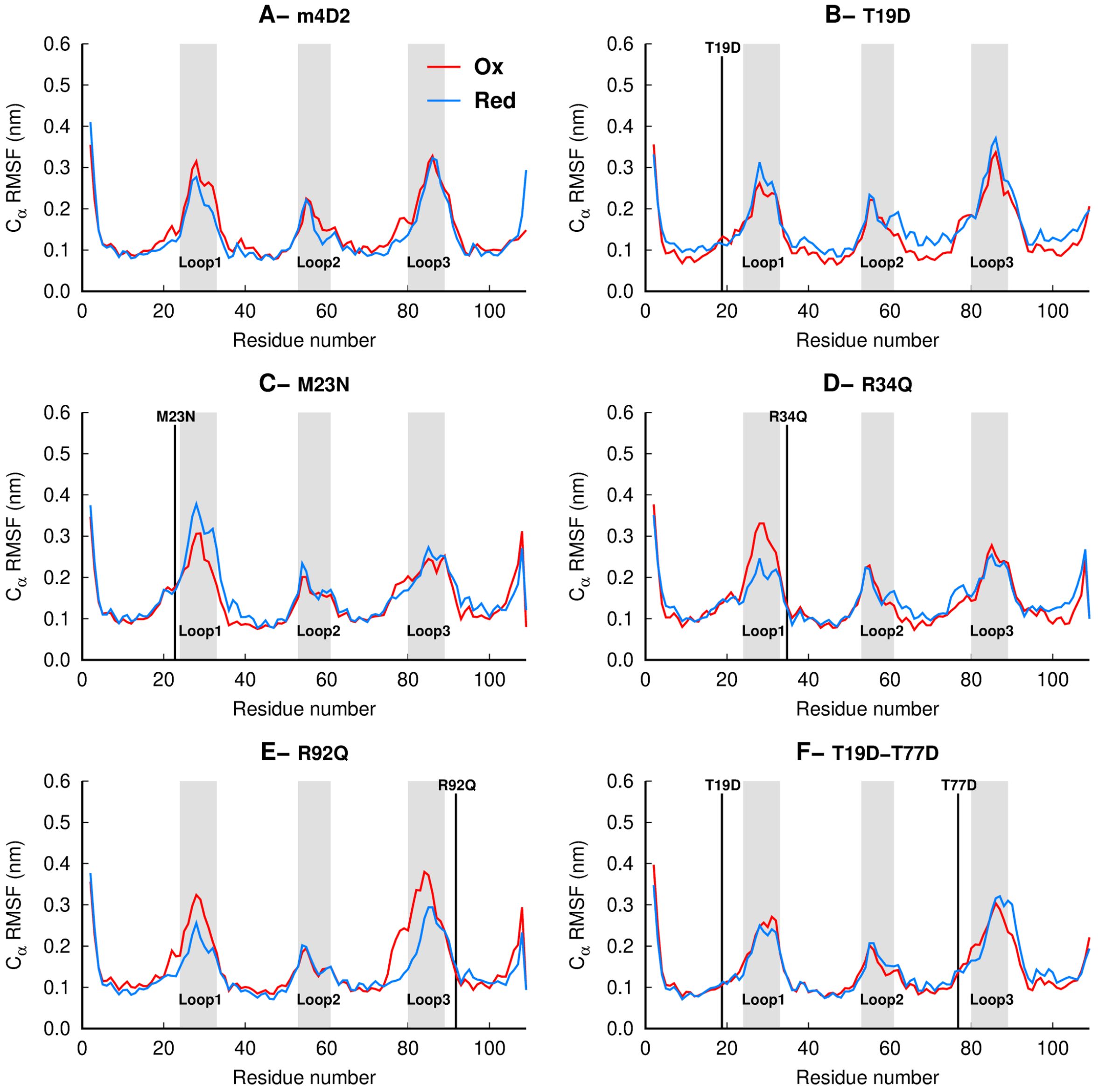

Figure S8: Average Cα RMSF for m4D2, T19D, M23N, R34Q, R92Q and T19D-T77D. The Cα RMSF was calculated for the last 400 ns of simulation and averaged over all replicates (10 replicates for m4D2 and single mutants and 20 replicates for the double mutant). The vertical black lines highlight the mutation site(s). The grey boxes identify the position of the loop regions, namely loop 1 (connecting α-helix 1 to α-helix 2), loop 2 (connecting α-helix 2 to α-helix 3) and loop 3 (connecting α-helix 3 to α-helix 4). Please zoom into the image for a detailed visualisation.

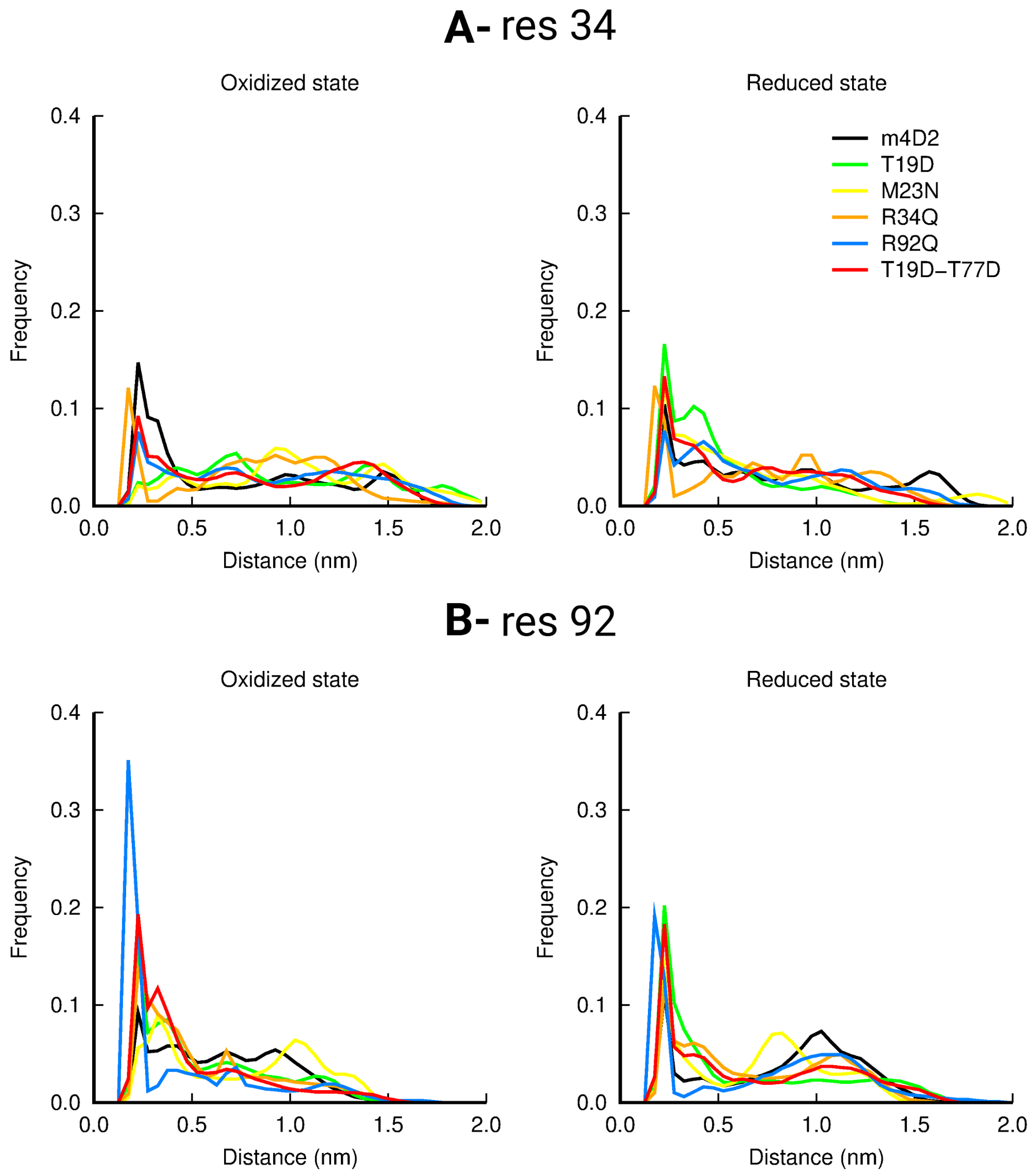

Figure S9: Minimum distance between residue 34 and the heme propionates (**A**) and residue 92 and the heme propionates (**B**) in the MD simulations of m4D2, T19D, M23N, R34Q, R92Q and T19D-T77D. Overall distribution of the minimum distance between the sidechain of residue 34 and 92 and the propionates. The histograms reflect the distances over the last 400 ns of each simulation.

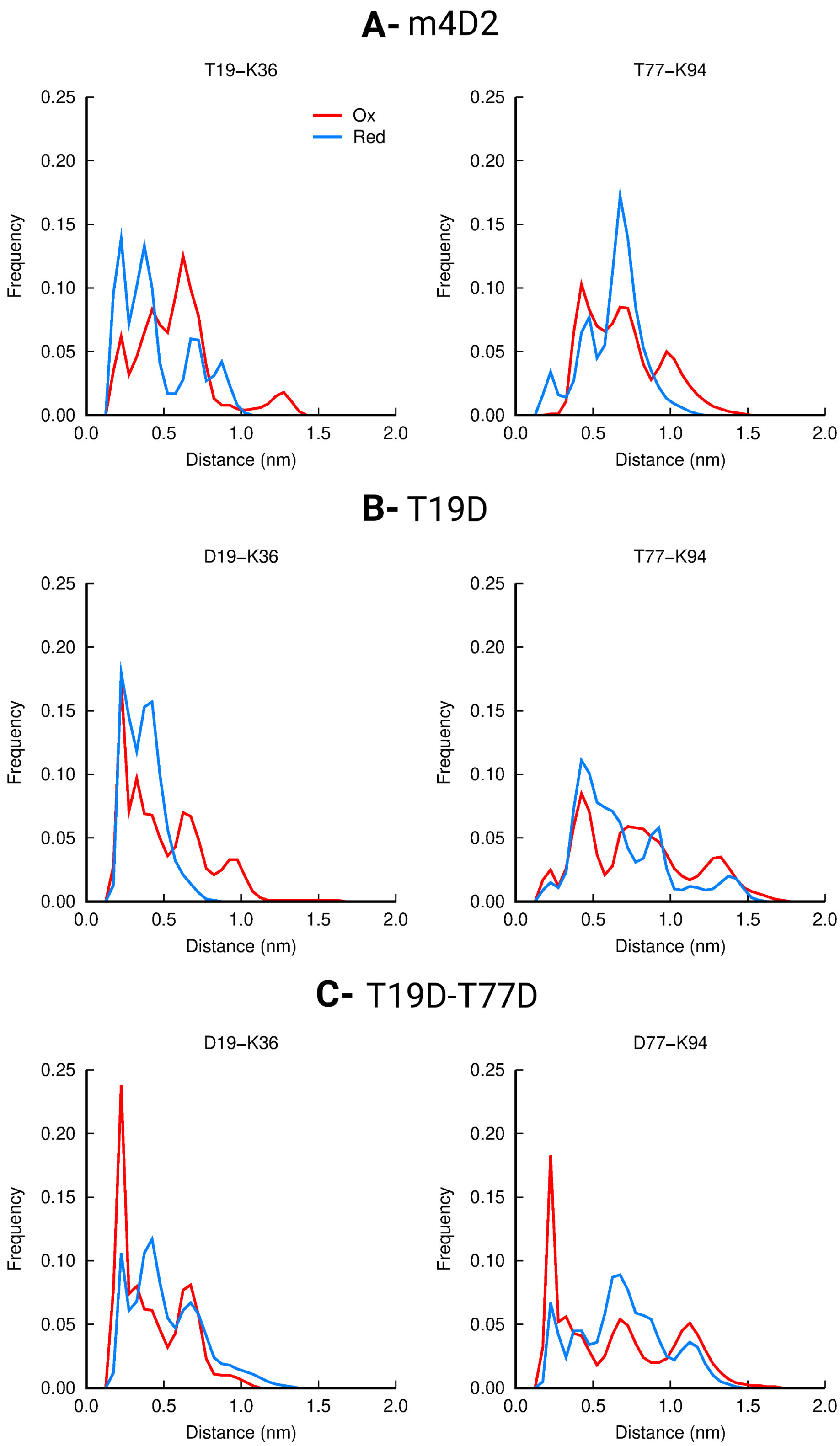

Figure S10: Minimum distance between residue in position 19 and 36, and 77 and 94 for (**A**) m4D2, (**B**) T19D and (**C**) T19D-T77D. Overall distribution of the minimum distance between the sidechain of residue 19 and 36, and 77 and 94. The histograms reflect the distances over the last 400 ns of each simulation.

S.2 Continuum electrostatics calculations

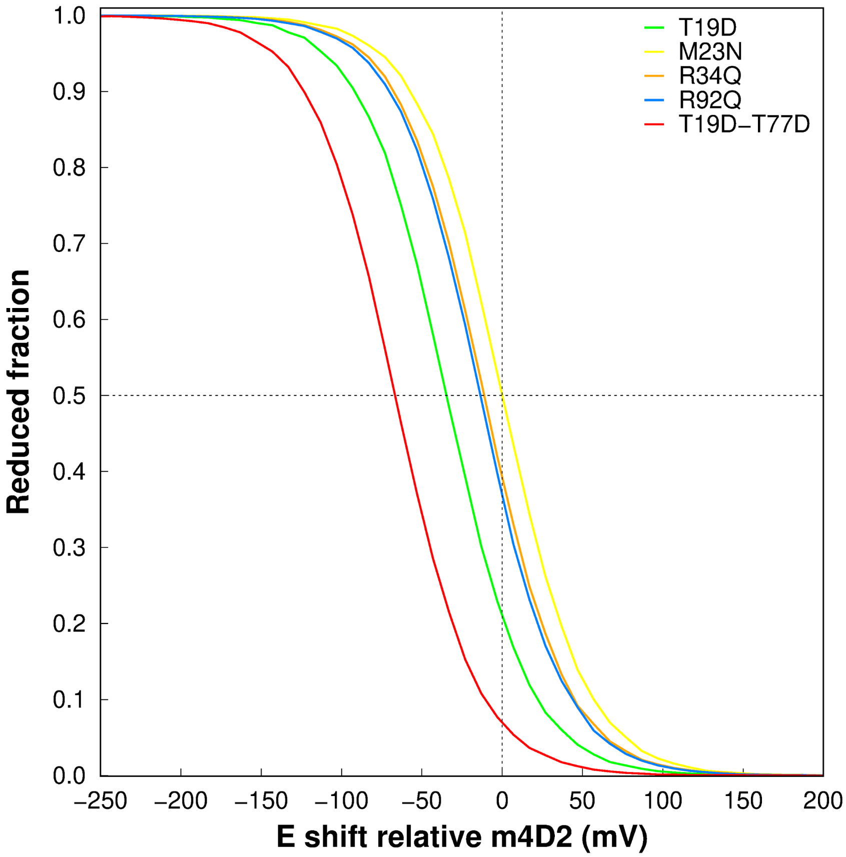

Figure S11: Individual reduction curve for T19D, M23N, R34Q, R92Q and T19D-T77D relative to m4D2. These curves were obtained using the PB+MC method, a combination of Poisson-Boltzmann (PB) calculations and Monte Carlo (MC) simulations.[2]

S.3 Theory of estimation of free energy differences using the Crooks-Bayes method

Consider a protocol Λ_i connecting two thermodynamic states, A_i and B_i, whose difference in free energy is $\Delta G_{\Lambda_i} = G_{B_i} - G_{A_i}$. Let W_i be the work cost of implementing such a protocol. Maragakis *et al.*[3] showed that, in the event of implementing $i = 1, \ldots, \nu$ of these protocols, the probability density $p(\boldsymbol{g}|\boldsymbol{W}, \boldsymbol{\Lambda})$ for the vector of hypotheses $\boldsymbol{g} = (g_{A_1}, g_{B_1}, \ldots, g_{A_\nu}, g_{B_\nu})$ about the true free energies $\boldsymbol{G} = (G_{A_1}, G_{B_1}, \ldots, G_{A_\nu}, G_{B_\nu})$, given the work vector $\boldsymbol{W} = (W_1, \ldots, W_\nu)$ and the protocol vector $\boldsymbol{\Lambda} = (\Lambda_1, \ldots, \Lambda_\nu)$, can be taken as

$$p(\boldsymbol{g}|\boldsymbol{W}, \boldsymbol{\Lambda}) \propto \prod_{i=1}^{\nu} f(\beta W_i - \beta \Delta g_{\Lambda_i} + M_{\Lambda_i}), \tag{S1}$$

where β is an inverse temperature, $f(x) = 1/[1 + \exp(-x)]$ is the logistic function, and each M_{Λ_i} is defined as

$$M_{\Lambda_i} = \log \left[\frac{p(\Lambda_i|\Delta g_{\Lambda_i})}{p(\tilde{\Lambda}_i|\Delta g_{\tilde{\Lambda}_i})} \right]. \tag{S2}$$

Here, $\Delta g_{\tilde{\Lambda}_i} = -\Delta g_{\Lambda_i}$ and $\tilde{\Lambda}_i$ labels a backward protocol connecting B_i and A_i.

Eq. (S1) rests on four pillars:[3] (i) Bayes theorem, written as $p(\Delta g_{\Lambda_i}|W_i, \Lambda_i) \propto p(\Delta g_{\Lambda_i})\, p(W_i, \Lambda_i|\Delta g_{\Lambda_i})$, where $p(\Delta g_{\Lambda_i})$ and $p(\Delta g_{\Lambda_i}|W_i, \Lambda_i)$ are the prior and posterior probabilities, respectively, and $p(W_i, \Lambda_i|\Delta g_{\Lambda_i})$ is the likelihood function; (ii) an uninformed state of information about both ΔG_{Λ_i} and the work distribution associated with each protocol, represented by $p(\Delta g_{\Lambda_i}) \propto 1$ and $p(W_i, \Lambda_i|\Delta g_{\Lambda_i}) + p(-W_i, \tilde{\Lambda}_i|\Delta g_{\tilde{\Lambda}_i}) \propto 1$, respectively; (iii) Crooks' relation[3–5]

$$\frac{p(W_i|\Delta g_{\Lambda_i}, \Lambda_i)}{p(-W_i|\Delta g_{\tilde{\Lambda}_i}, \tilde{\Lambda}_i)} = \mathrm{e}^{\beta(W_i - \Delta g_{\Lambda_i})}, \tag{S3}$$

which is a detailed fluctuating work symmetry; and (iv) uncorrelated work values. We thus refer to the application of Eq. (S1) as the Crooks-Bayes method. Assumptions (ii - iv) encapsulate the information available prior to collecting the simulated work data. We further note that, unlike in the main text, the work probabilities in Eq. (S3) are written as conditional on the free energy difference. This is a matter of notation; in the main text, Crooks' relation is first discussed in the context of histogram-based estimation, where the aforementioned conditioning can be ignored. This cannot however be done when employing Bayesian estimation.[6]

To predict redox potentials via Eq. (S1), we first need to adapt it to our particular scenario, as follows. Imagine $\nu = 2\mu$ experiments such that half of them correspond to the repetitions of a single protocol, connecting the thermodynamic states A and B, while the the other half corresponds to repeating the backward protocol, i.e., connecting B and A. In that case, the vector of free energies is given as

$$\boldsymbol{G} = (\underbrace{G_A, G_B, \ldots, G_A, G_B}_{\mu \text{ times}}, \underbrace{G_B, G_A, \ldots, G_B, G_A}_{\mu \text{ times}}).$$

By defining $\Delta G \equiv G_B - G_A$, we further see that $\Delta G_{\Lambda_i} = \Delta G$ for all i. It is then appropriate to rename $p(\boldsymbol{g}|\boldsymbol{W}, \boldsymbol{\Lambda})$ as $p(\Delta g|\boldsymbol{W}, \boldsymbol{\Lambda})$, where, in this case, $\boldsymbol{W} = (W_1, \ldots, W_{2\mu})$ and $\boldsymbol{\Lambda} = (\Lambda_1, \ldots, \Lambda_\mu, \tilde{\Lambda}_{\mu+1}, \ldots, \tilde{\Lambda}_{2\mu})$. The fact that we have the same number of forward and backward iterations further allows each M_{Λ_i} to be approximated as $M_{\Lambda_i} \approx 0$.[3] Putting all the pieces together, Eq. (S1) becomes

$$\begin{aligned} p(\Delta g|\boldsymbol{W}, \boldsymbol{\Lambda}) &\propto \prod_{i=1}^{\mu} f(\beta W_i - \beta \Delta g_{\Lambda_i}) \prod_{j=\mu+1}^{2\mu} f(\beta W_j - \beta \Delta g_{\tilde{\Lambda}_i}) \\ &= \prod_{i=1}^{\mu} f(\beta W_i - \beta \Delta g) \prod_{j=\mu+1}^{2\mu} f(\beta W_j + \beta \Delta g). \end{aligned} \tag{S4}$$

To simplify the notation, we may now drop the dependence on the protocol vector as $p(\Delta g|\boldsymbol{W}, \boldsymbol{\Lambda}) \mapsto p(\Delta g|\boldsymbol{W})$. Inserting the work values $\boldsymbol{W}$ associated with the protocols $\boldsymbol{\Lambda}$, where $W_i = \epsilon^i_{\text{red}} - \epsilon^i_{\text{ox}} = \Delta\epsilon_i$ for $i = 1, ..., \mu$, and $W_i = \epsilon^i_{\text{ox}} - \epsilon^i_{\text{red}} = \Delta\epsilon_i$ for $i = \mu+1, ..., 2\mu$, in Eq. (S4), produces Eq. (2) in the main text. This allows us to calculate the likelihood of different hypotheses Δg, as shown in Fig. S14 for the oxidation and reduction of the heme group. This then leads to the redox potential estimates reported in the main text, here shown in Fig. S15.

S.4 Estimation of redox potentials from non-acknowledgement work data

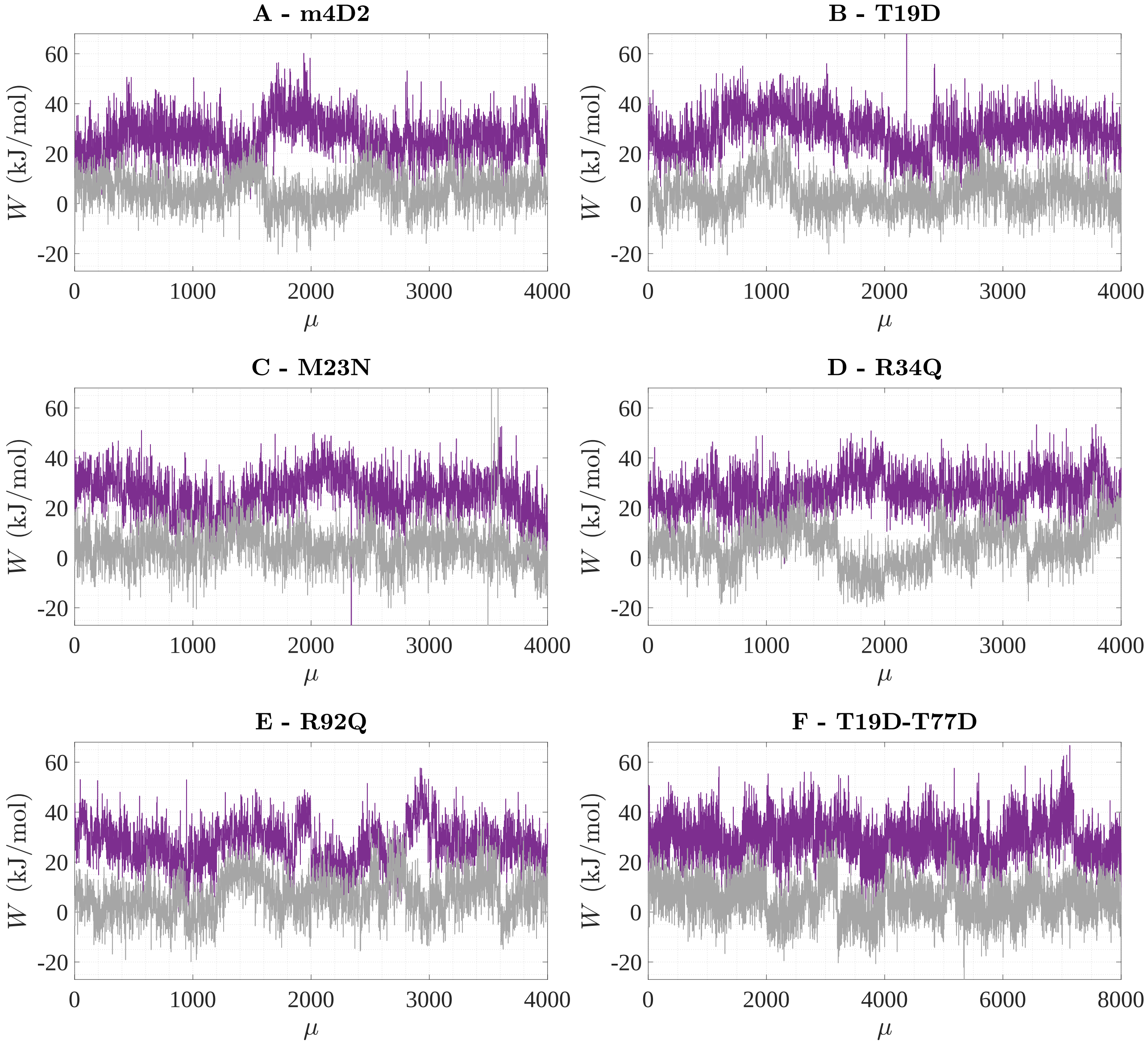

Figure S12: Statistical work values W and $-W$ for the forward/reduction (purple) and backward/oxidation (grey) processes, respectively. Each W is given as $W = \Delta\epsilon$ where $\Delta\epsilon$ denotes the change of energy of the protein[5,7].

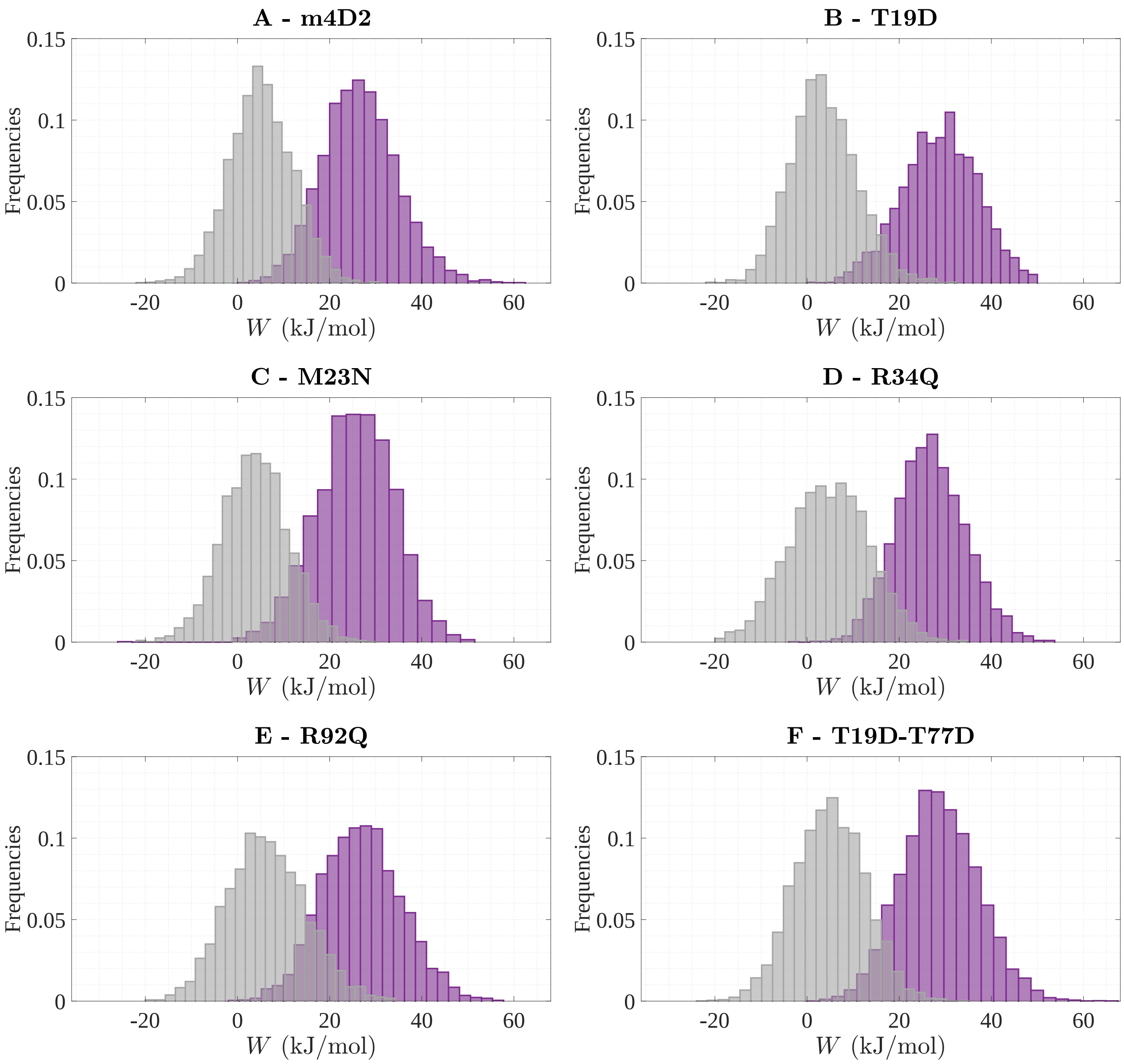

Figure S13: Work histograms $p(W|\Lambda)$ and $p(-W|\tilde{\Lambda})$ for the forward/reduction (Λ; purple) and backward/oxidation ($\tilde{\Lambda}$; grey) processes obtained from the data in Fig. S12, respectively. These have been generated using the `histogram` function in MATLAB. Note that the Crooks-Bayes method discussed in Sec. S.3 does not make use of these histograms, which are shown here for illustrative purposes only. While histogram-based estimation coupled with statistical bootstrapping is the current standard in the literature, we found that such a method was less precise and computationally slower for a given data set, thus justifying our choice of using the Crooks-Bayes approach in this work.

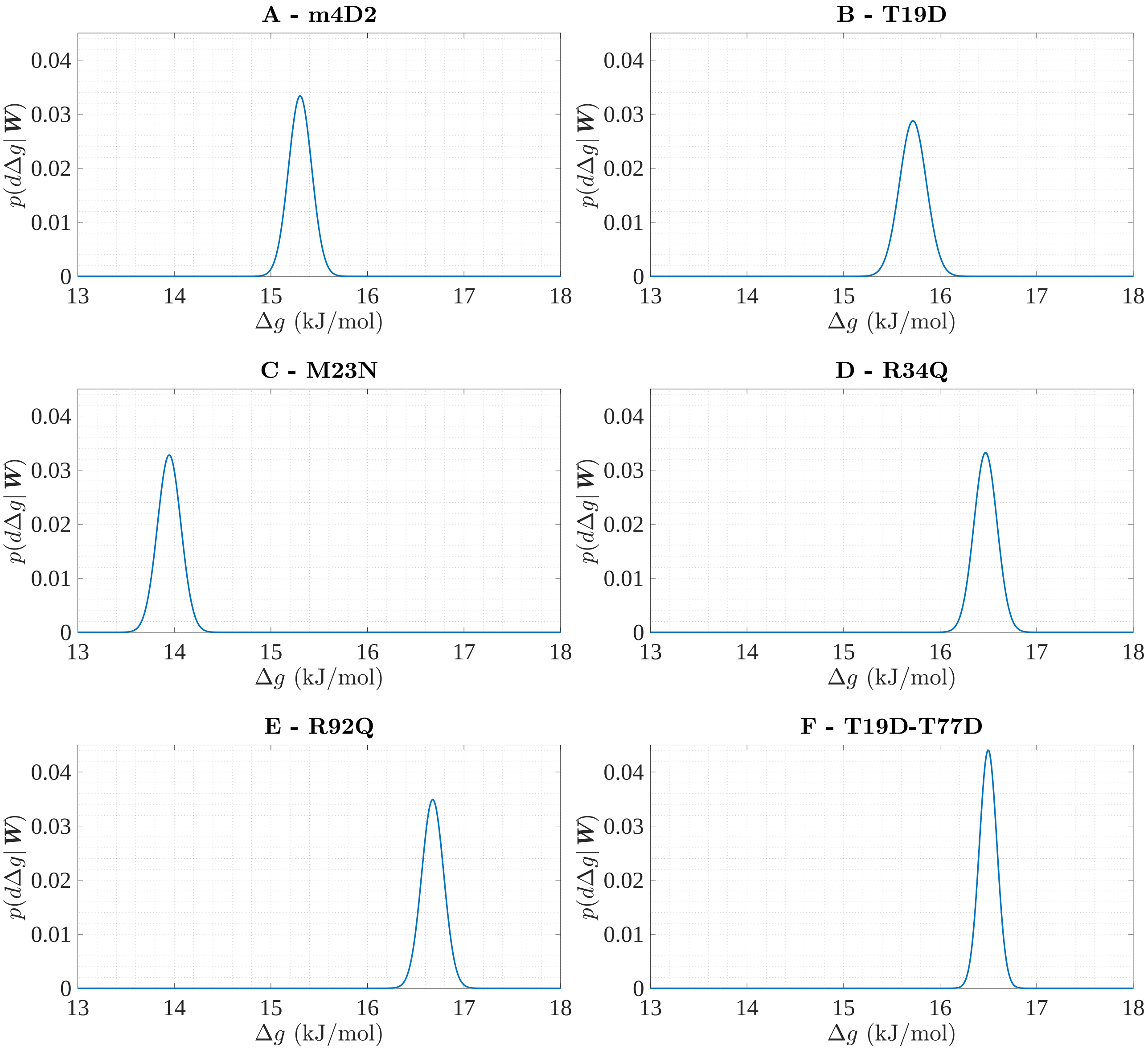

Figure S14: Numerical posterior probability $p(d\Delta g|\boldsymbol{W}) = p(\Delta g|\boldsymbol{W})d\Delta g$ calculated via Eq. (S4) using the work data $\boldsymbol{W}$ in Fig. S12. This amounts to multiplying a large number of logistic functions with their mirror image, thus leading to the bell-like shape that can be observed.[3] More importantly, each profile encodes all the available information needed to calculate an estimate for the true free energy difference ΔG. To normalise these profiles, we chose a conservative range of $\Delta g \in [-396, 531]$ kJ/mol, which covered the most likely work values produced by our MD simulations. As shown in Fig. S15, these estimates enable the means to predict redox potentials E, given the relation $E = -\Delta G/F$. Here, E and ΔG are measured in units of voltage and of energy, respectively, while F stands for Faraday's constant.

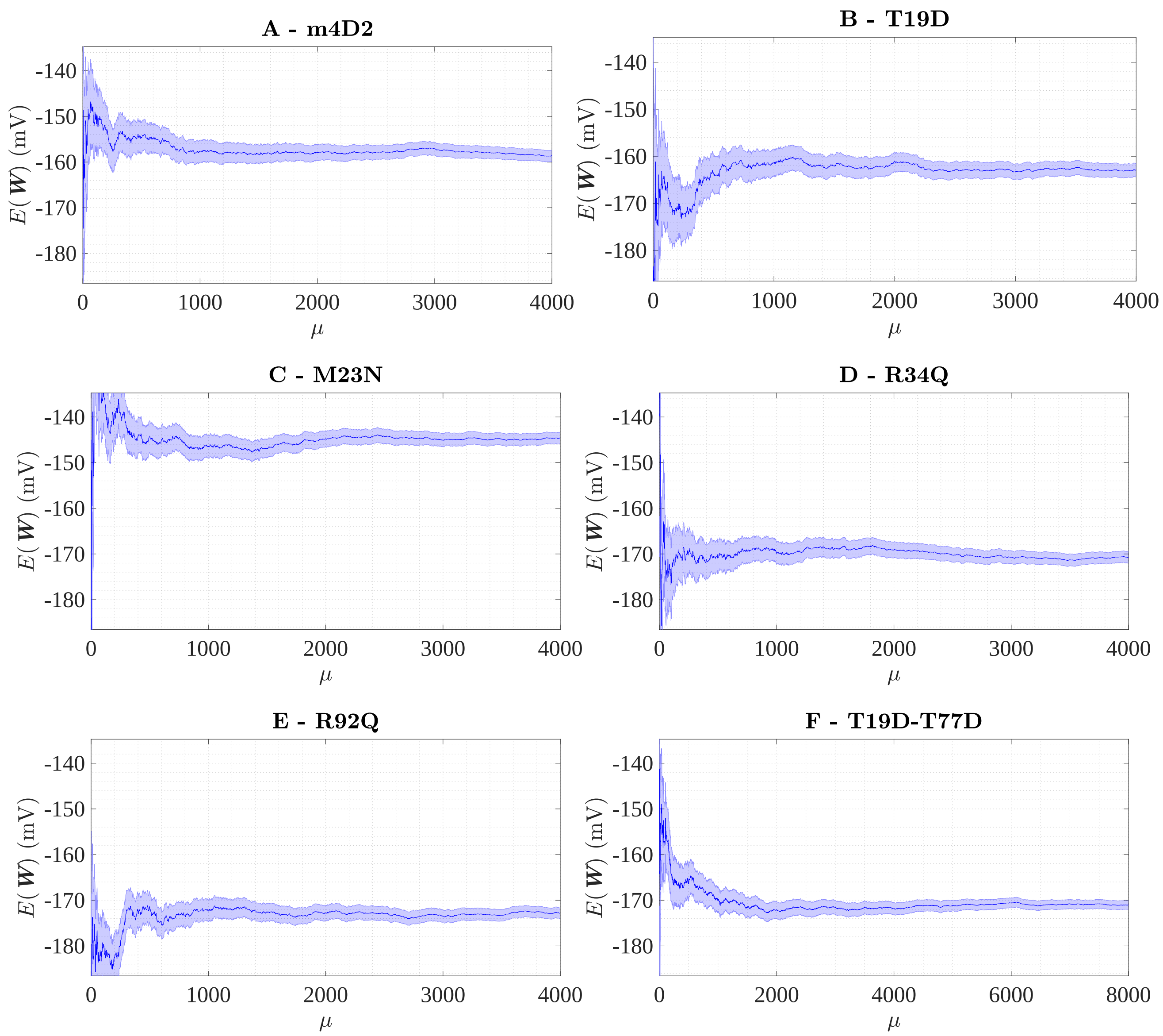

Figure S15: Crooks-Bayes estimates $\tilde{E}(\boldsymbol{W}) \pm \sigma(\boldsymbol{W})$ from μ iterations of the forward/reduction and backward/oxidation protocols. These are calculated using the estimator $E(\boldsymbol{W}) = -\int d\Delta g\, p(\Delta g|\boldsymbol{W})\Delta g/F$, which is optimal under the square error criterion.[6,8] Here, F is Faraday's constant. The error in such an estimate is given by $\sigma^2(\boldsymbol{W}) = \int d\Delta g\, p(\Delta g|\boldsymbol{W})[-\Delta g/F - E(\boldsymbol{W})]^2$. The posterior probabilities $p(\Delta g|\boldsymbol{W})$ are those in Fig. S14. The final values for the redox potentials in the main text correspond to $\mu = 4000$ iterations in panel A-E, and to $\mu = 8000$ iterations in panel F. As can be observed, all six estimates start to converge to a single value when $\mu \simeq 2000$.

S.5 Experimental measurement of the redox potentials

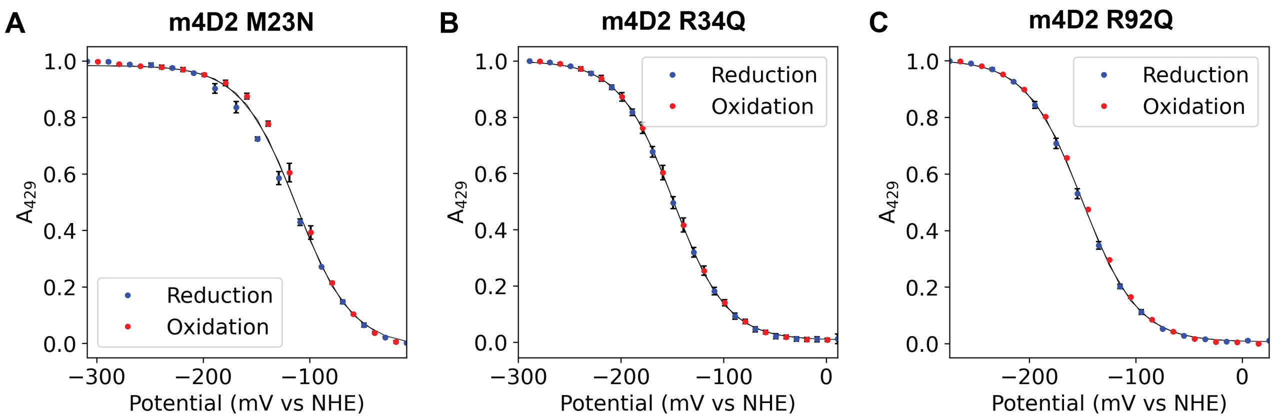

Figure S16: Experimental redox potentiometry of M23N, R34Q and R92Q recorded in 20 mM CHES, 100 mM KCl, 10% glycerol, pH 8.6. Data were fitted to a single electron Nernst function.

S.6 Experimental vs predicted redox potentials

Table S1: Experimental (column 2) and predicted (column 3) redox potentials E for m4D2, T19D, M23N, R34Q, R92Q and the double mutant T19D-T77D (DM). These correspond to the methods employed in Fig. S16 and Fig. S15, respectively. The predicted redox potentials, in particular, were calculated using the proposed MD+CB method, which post-processes the data generated by the MD simulations via the Crooks-Bayes estimator as described in the main text (Sec.2.4) as well as here (Secs. S.3 and S.4). The errors associated with E are shown in parentheses. Previously measured redox potentials[9] are referenced in the first column.

protein	E **experiment** (mV)	E **predicted** (mV)
m4D2[9]	-118 (1)	-159 (1)
T19D[9]	-146 (1)	-163 (1)
M23N	-119 (1)	-145 (1)
R34Q	-149 (1)	-171 (1)
R92Q	-150 (1)	-173 (1)
DM[9]	-174 (1)	-171 (1)

References

(1) Kabsch, W.; Sander, C. Dictionary of protein secondary structure: Pattern recognition of hydrogen-bonded and geometrical features. *Biopolymers* **1983**, *22*, 2577–2637.

(2) Teixeira, V. H.; Soares, C. M.; Baptista, A. M. Studies of the reduction and protonation behavior of tetraheme cytochromes using atomic detail. *Journal of Biological Inorganic Chemistry* **2002**, *7*, 200–216.

(3) Maragakis, P.; Ritort, F.; Bustamante, C.; Karplus, M.; Crooks, G. E. Bayesian estimates of free energies from nonequilibrium work data in the presence of instrument noise. *The Journal of Chemical Physics* **2008**, *129*, 024102.

(4) Crooks, G. E. Entropy production fluctuation theorem and the nonequilibrium work relation for free energy differences. *Physical Review E* **1999**, *60*, 2721–2726.

(5) Seifert, U. Stochastic thermodynamics, fluctuation theorems and molecular machines. *Reports on progress in physics* **2012**, *75*, 126001–126001.

(6) Jaynes, E. T. *Probability Theory: The Logic of Science*; Cambridge University Press, 2003.

(7) Jarzynski, C. Nonequilibrium Equality for Free Energy Differences. *Physical Review Letters* **1997**, *78*, 2690–2693.

(8) von Toussaint, U. Bayesian inference in physics. *Reviews of Modern Physics* **2011**, *83*, 943–999.

(9) Hutchins, G. H.; Noble, C. E. M.; Bunzel, H. A.; Williams, C.; Dubiel, P.; Yadav, S. K. N.; Molinaro, P. M.; Barringer, R.; Blackburn, H.; Hardy, B. J.; Parnell, A. E.; Landau, C.; Race, P. R.; Oliver, T. A. A.; Koder, R. L.; Crump, M. P.; Schaffitzel, C.; Oliveira, A. S. F.; Mulholland, A. J.; Anderson, J. L. R. An expandable, modular de novo protein platform for precision redox engineering. *Proceedings of the National Academy of Sciences* **2023**, *120*, e2306046120.
